# Supplementary material for: Allosteric regulation and crystallographic fragment screening of SARS-CoV-2 NSP15 endoribonuclease
Source: Nucleic Acids Res. 2023 Apr 28;51(10):5255–70. doi: 10.1093/nar/gkad314 (PMC10250223; doi:10.1093/nar/gkad314)
Supplement: gkad314_Supplemental_Files [file gkad314_supplemental_files.zip › Supplementary datasets.zip!/81/Refine_9/01-BUSTER/Cycle-4/LIST.html]

Refining

**BUSTER 2.X**: A suite of programs for macromolecular structure refinement by maximum-likelihood and Bayesian methods  
Copyright © 1992-2020 Global Phasing Ltd.  
All rights reserved.  
Authors: G. Bricogne, E. Blanc, M. Brandl, C. Flensburg, P. Keller, W. Paciorek, P. Roversi, O. Smart, C. Vonrhein, T. Womack  

Please reference use of the software as:

- BUSTER-TNT 2.X, Global Phasing Ltd, Sheraton House, Cambridge CB3 0AX, UK

Version: 2.10.3 for linux64-ifort made on Sep 18 2020 at 01:21:52  
Licenced to: Diamond Light Source, Scientific Software  

```
---


### Parsing of the BUSTER cardfile

TITLE : autoBUSTER refinement

   Cell information

   Symmetry information

 Using CCP4 X-ray form factors.

   Missing structure chemical composition

 Using ATOM block for chemical composition.


   Shell information

### Parsing of the TNT cardfile


---


### Reading observations file

 Number of reflexions in input:  57108

      Number of accepted reflexions:  55835

      Number of rejected reflexions:      0

   Reflexions in test set:            2859

   Reflexions in working set:        52976


---


### 1. Generating initial model

 2.10 * Shannon sampling rate. NX NY NZ:   288  288  240


  Effective Shannon rates are:    2.29    2.29    2.67


   Allocated BUFFER of size:     76.465 Mb.

   Initial Geometry Information


 No missing atoms declared


   Summary of model volumes

 No missing atoms declared: no prior computed


 Computing bulk solvent model scattering


 NCSMASK PDB radius for solvent mask:     2.15 Angstrom

 running PDBSET (setting cell and spacegroup P1) ... done
 running NCSMASK [1] (creating mask, SMOOTH 1.05084) ... done
 running MAPDUMP (to check if reduction to unit cell is needed) ... done
 running MAPMASK (converting mask to map) ... done

   Generation of babslv by theta-filter

   Electron density in frg mask:  0.420 Å-1

 FOBS  put on rough absolute scale by Wilson plot :
           K        =  0.46454E+02
           B        =    -18.483

 

### Cycle 0001: MaxLik scaling and refinement


   MaxLik Scaling Cycle 0001

   Wilson plot

   Statistics in resolution bins just after scaling
```

|  |  |
| --- | --- |
|  | - Rxpct factors plot- Rxpct factors table- <Log-Lik Gain>- Ln Sigma A- Correlation Coefficients |

```
   <Log-Likelihood> of null-hypothesis

Structure Factor Amplitudes


   Average Figure of Merit

 Switching off refinement of overall temperature factor


 Added B =     0.5756 Å2 to all atoms in fragment


 Added B =     0.5756 Å2 to B_SOLV

```
gelly refinement module
 Authors (2004-2009) O. S. Smart, C. Flensburg, W. Paciorek, C. Vonrhein, 
                     M. Brandl, T. O. Womack and G. Bricogne
         (2010-2011) O. S. Smart, C. Flensburg, W. Paciorek, C. Vonrhein, 
                     T. O. Womack and G. Bricogne
         (2012-2014) O. S. Smart, C. Flensburg, W. Paciorek, C. Vonrhein, 
                     and G. Bricogne
         (2015-2020) G. Bricogne, C. Flensburg, W. Paciorek, C. Vonrhein
 Copyright © 2004-2020 Global Phasing Ltd. 
 All rights reserved.
 gelly module compilation date Sep 18 2020 01:18:35
 
 gelly uses routines from TNT geometry. References: 
     Tronrud, D. E., Ten Eyck, L. F., Matthews, B. W. (1987)
     "An Efficient General-Purpose Least-Squares Refinement Program 
     for Macromolecular Structures". Acta Crystallogr A, 43:489-501.
     Tronrud, D. E. (1997). "The TNT Refinement Package", 
     in Macromolecular Crystallography, Part B, Eds Charlie Carter 
     and Robert Sweet, Methods in Enzymology, 277:306-319.
 wildcard matching function by Julian Robichaux http://www.nsftools.com

 No -random_seed flag found. Random number stream initialised using:
2147483562
2147483398

 Control flags set to:  -autoncs -hydrogens_input_occ2zero \
                     -hydrogens_output_occ2heavy -screen \
                     100 -screen_sigma \
                     3.0 -keeppdb \
                     1000000 -taomonitor_verbose \
                     2 
 

 ############### Starting setup via TNT routines

 Setting up tnt routines including license check:

                   TNT Stereochemistry Program
 Version: 5.22.3.0
 Licence for Diamond Light Source, Scientific Software is valid
 
 Using TNT geometry routines to load the tnt control file: gelly.tnt
 Using TNT routines to load initial coordinates from file fragment.cor
 Expecting 5526 atoms

 Found     2171 "NOTE BUSTER_RESIDUE_ATOM_DATA" cards
 Have copied information for    5526 atoms from TNT to gelly's store 
 
 The TNT WEIGHT cards used by this run:
 -----WEIGHT       BOND     2.0
 -----WEIGHT       ANGLE    2.0
 -----WEIGHT       IMPROPER 0.0
 -----WEIGHT       TORSION  2.0
 -----WEIGHT       PSEUDO   0.0
 -----WEIGHT       TRIGONAL 2.0
 -----WEIGHT       PLANE    5.0
 -----WEIGHT       CONTACT  5.0
 -----WEIGHT       BCORREL  10.0
 -----WEIGHT       CHIRAL   5.0
 -----WEIGHT       NCS      50.0
 -----WEIGHT       IDEAL    4.0
 -----WEIGHT       RFACTOR  7.24
 
 Dictionary versions picked up from NOTE BUSTER_RESTRAINT_DICT cards as: 
 ------ protgeo_eh99.dat (V1.6.4.17) 20191001  STANDARD AMINO ACID DICTIONARY. BONDS AND ANGLES FROM ENGH AND HUBER EH99. OTHER VALUES BASED ON PREVIOUS TNT OR TAKEN FROM CCP4. INCLUDES HYDROGEN ATOMS.
 ------ exoticaa.dat (V1.3.4.15) 20200327  COLLECTION OF NON-STANDARD AMINO ACIDS, MAINLY EH91 WITHOUT IDEAL DISTANCE INFO
 ------ nuclgeo.dat (V1.13.4.8) 20190909
 ------ bcorrel.dat (V1.15.10.2) 20190616
 ------ contact.dat (V1.15.12.10) 20191121
 ------ idealdist_contact.dat (V1.3.4.12) 20191001  IDEAL-DISTANCE CONTACT TERM DATA AS USED IN PROLSQ. VALUES USED HERE ARE BASED ON DATA HARVESTING OF PDB AS OF 20190109.
 ------ restraints for LIG (Unknown) from cif dictionary Z1741794237.cif
 ------ assume.dat (V1.9.4.2) 20180705
 
 Have picked up spacegroup name for pdb output as 'P 63      '
 Setting up Chain/Residue arrays
 
 Have command line specification -autoncs*
     Have found       1 NCS relations
     Pairwise matching residue number and type between chains: 
                    A        B    
          A         .      100%  
          B                  .   
       Table key--                                                       
          all   : two chains have 100% match and same number of residues 
         multi  : ditto and they contribute to a multi chain ncs cluster 
          ***%  : two chains have >80% matching residue number/type      
           -    : chains appear to be unrelated                          
      chain A     have identified ncs to     1 other chains. So will weight each autoncs by (       6.666/#)=      6.6660
      chain B     have identified ncs to     1 other chains. So will weight each autoncs by (       6.666/#)=      6.6660
     For .Gelly cards produced see below - after pruning
 
 Setting up BUSTER_SET groups
 Have found    89 BUSTER_SET cards
 Now silently applying automatic/dictionary set definitions. For a verbose listing use '-verbose_set' flag.
 Applying user defined set definitions:
 =====NOTE BUSTER_SET ncsautoXcld = Water --> set 'ncsautoXcld' now has     0 atoms 
 =====NOTE BUSTER_SET ncsautoABset = Chain_A \ ncsautoXcld --> set 'ncsautoABset' now has  2768 atoms 
 Have defined    47 sets: 
      set:                 'All' has   5526 atoms in    699 residues - automatically defined
      set:               'Empty' has      0 atoms in      0 residues - automatically defined
      set:            'ExoticAA' has      0 atoms in      0 residues - defined by dictionary file
      set:           'ExoticNUC' has      0 atoms in      0 residues - defined by dictionary file
      set:              'NotHET' has   5516 atoms in    698 residues - defined by dictionary file
      set:             'Chain_A' has   2768 atoms in    350 residues - automatically defined
      set:             'Chain_B' has   2758 atoms in    349 residues - automatically defined
      set:               'Water' has      0 atoms in      0 residues - defined by dictionary file
      set:          'StdProtein' has   5516 atoms in    698 residues - defined by dictionary file
      set:             'Protein' has   5516 atoms in    698 residues - defined by dictionary file
      set:                'Back' has   2794 atoms in    698 residues - defined by dictionary file
      set:                'Side' has   2722 atoms in    652 residues - defined by dictionary file
      set:        'RnaCanonical' has      0 atoms in      0 residues - defined by dictionary file
      set:        'DnaCanonical' has      0 atoms in      0 residues - defined by dictionary file
      set:     'RnaDnaCanonical' has      0 atoms in      0 residues - defined by dictionary file
      set:     'DnaRnaCanonical' has      0 atoms in      0 residues - defined by dictionary file
      set:         'RnaExtended' has      0 atoms in      0 residues - defined by dictionary file
      set:         'DnaExtended' has      0 atoms in      0 residues - defined by dictionary file
      set:      'RnaDnaExtended' has      0 atoms in      0 residues - defined by dictionary file
      set:      'DnaRnaExtended' has      0 atoms in      0 residues - defined by dictionary file
      set:                 'Dna' has      0 atoms in      0 residues - defined by dictionary file
      set:                 'Rna' has      0 atoms in      0 residues - defined by dictionary file
      set:              'RnaDna' has      0 atoms in      0 residues - defined by dictionary file
      set:              'DnaRna' has      0 atoms in      0 residues - defined by dictionary file
      set:             'Nucleic' has      0 atoms in      0 residues - defined by dictionary file
      set:             'Polymer' has   5516 atoms in    698 residues - defined by dictionary file
      set: 'BufferWeichenberger' has      0 atoms in      0 residues - defined by dictionary file
      set:    'BufferAdditional' has      0 atoms in      0 residues - defined by dictionary file
      set:              'Buffer' has      0 atoms in      0 residues - defined by dictionary file
      set:         'MetalAlkali' has      0 atoms in      0 residues - defined by dictionary file
      set:  'MetalAlkalineEarth' has      0 atoms in      0 residues - defined by dictionary file
      set:     'MetalTransition' has      0 atoms in      0 residues - defined by dictionary file
      set: 'MetalPostTransition' has      0 atoms in      0 residues - defined by dictionary file
      set:    'MetalLanthanides' has      0 atoms in      0 residues - defined by dictionary file
      set:               'Metal' has      0 atoms in      0 residues - defined by dictionary file
      set:         'SugarAlphaD' has      0 atoms in      0 residues - defined by dictionary file
      set:         'SugarAlphaL' has      0 atoms in      0 residues - defined by dictionary file
      set:               'Sugar' has      0 atoms in      0 residues - defined by dictionary file
      set:               'Other' has     10 atoms in      1 residues - defined by dictionary file
      set:            'Hydrogen' has      0 atoms in      0 residues - defined by dictionary file
      set:         'nonHydrogen' has   5526 atoms in    699 residues - defined by dictionary file
      set:             'OccZero' has      0 atoms in      0 residues - defined by dictionary file
      set:          'OccNonZero' has   5526 atoms in    699 residues - defined by dictionary file
      set:             'OccFull' has   5484 atoms in    696 residues - defined by dictionary file
      set:          'OccNonFull' has     42 atoms in      3 residues - defined by dictionary file
      set:         'ncsautoXcld' has      0 atoms in      0 residues - user specified
      set:        'ncsautoABset' has   2768 atoms in    350 residues - user specified
 No command line arguments '-occ_allhet' or '-occ_restyp' so will not setup automated occcupancy refinement
 
 Interpreting CONSTANT and FREE cards: 
 =====CONSTANT     OCC
 Have found and interpreted      1 CONSTANT cards in the TNT input. 
 Before any COMBINE - Number of atoms with fixed XYZ =     0
 Before any COMBINE - Number of atoms with fixed B   =     0
 Before any COMBINE - Number of atoms with fixed OCC =  5526
 Before any COMBINE - Number of atoms with fixed BIJ =     0
                            cf total number of atoms =  5526
 
 There are no COMBINE cards to be found in the TNT card input 
 
 There are no NOTE BUSTER_COMBINE_RES cards to be found in the TNT card input 

 ############### Working out full connectivity list


 ############### Working out equivalent atoms


 Residue with most atoms given below:
        A|58:N(TRP)         
        A|58:CA(TRP)        
        A|58:CB(TRP)        
        A|58:CG(TRP)        
        A|58:CD1(TRP)       
        A|58:NE1(TRP)       
        A|58:CE2(TRP)       
        A|58:CD2(TRP)       
        A|58:CE3(TRP)       
        A|58:CZ3(TRP)       
        A|58:CH2(TRP)       
        A|58:CZ2(TRP)       
        A|58:C(TRP)         
        A|58:O(TRP)         


 Have no '-equiv_check_plus' argument - so will take atom type into
 account when checking for equivalence of otherwise identical atoms.
 

 ############### Looking at dihedrals


 ############### Looking at chirals

 NOTE BUSTER_CHIRAL_ANGLECUT card found - read value=    -31.00 degs from it
 NOTE BUSTER_CHIRAL_SIGMA card found - read value=      1.70 degs from it
 RANGE allowed for B variables in refinement:   from      3.00000 to    300.00000

 ############### Preparing for PDB output


 ############### Processing extra restraints explicitly defined

 No 'NOTE BUSTER_TARGET' cards or -target?? args found (used to read coordinates files for soft ncs to external)
 No 'NOTE BUSTER_OCCSUM'     cards found - so not using sum of occupancy restraints
 No 'NOTE BUSTER_DISTANCE'   cards found - so not setting up any additional atom-atom distance restraints
 No 'NOTE BUSTER_UTILANGLE'  cards found - so not setting up any additional restraints on any angle
 No 'NOTE BUSTER_UTILTOR'    cards found - so not setting up any additional sinusoidal restraints on torsions
 
 --- Have some similarity definitions 
     Similarity restraints on RMSD use superposition routines from: 
     Coutsias, E.A., Seok, C., Dill, K.A.(2004)
     "Using quaternions to calculate RMSD", J. Comput. Chem., 25:1849-1857 

 Have found    1 'NOTE BUSTER_SIM_DEFINE' cards 
     Maximum number of chains involved in any SIM group (excluding the template)=     1


 ########## Interpreting card: NOTE BUSTER_SIM_DEFINE ncsautoAB ncsautoABset B
     Number of atoms in template set=  2768
     Number of related chains (other than template) in SIM set=    1
     List of chains to be restrained (excluding template)=   B   
     Pairwise RMSD, XYZ in Angstroms, for SIM group: ncsautoAB
                 A         B   
     A         -----     ----- 
     B         0.626     ----- 
     Overall RMSD,XYZ for the SIM group is     0.626 Angstroms
     Pairwise rms deviation of [individual B's minus their set/chain average] in Angs**2
                 A         B         Bavg
     A         -----     -----     98.76
     B         38.13     -----     68.79
     Overall RMSD,B   for the SIM group is    38.134 Angs**2
     The number of common atoms for the above fits:
                 A         B   
     A         -----     ----- 
     B          2758     ----- 
     (compared to the template set that has    2768 atoms in it)
     List of atoms that are absent in one or more chains for this group: 
                                    A    B   
                    C01     401     +    -
                    C02     401     +    -
                    C03     401     +    -
                    N04     401     +    -
                    C05     401     +    -
                    C06     401     +    -
                    N07     401     +    -
                    C08     401     +    -
                    C09     401     +    -
                    O10     401     +    -
     (key  +  atom present for this chain 
           -  atom absent  for this chain)
 transforms for each of the superpositions orthogonal matrix r11 r12 r13 r21 r22 r23 r31 r32 r33 tx ty tz (PDBSET syntax): 
TRANS_AB -0.873154 -0.486753  0.025936 -0.486705  0.873524  0.008578 -0.026831 -0.005133 -0.999627    44.141   -12.857    15.132
 reverse transforms 
TRANS_BA -0.873154 -0.486705 -0.026831 -0.486753  0.873524 -0.005133  0.025936  0.008578 -0.999627    32.691    32.794    14.092
 
 Setting up LSSR interatomic distance restraints
 Interpreting card: NOTE BUSTER_SIM_RESTRAIN_LSSR ncsautoAB 6.666
        setting up interatomic distance pair restraints with:
        Dmin=         0.00
        Dmax=         5.50
        sigma=        0.20
        Vmax=         3.00
        weight=       6.67
        number of distance pairs defined for this set is      33845
 Have not supplied either '-sim_swap_equiv' or '-sim_swap_equiv_plus' argument 
   so will not try swaping equivalent atoms to improve NCS and/or target agreement.
 
 NCS by LSSR outlier analysis
 -autoncs option automatic NCS prune routine -------- 
 Residues pruned out by gradient condition:
           A|23      A|27      A|34      A|37      A|39     A|147     A|148
          A|149     A|150     A|151     A|158     A|159     A|168     A|169
          A|170     A|171     A|172     A|173     A|174     A|175     A|187
          A|188     A|216     A|217     A|220     A|229     A|259     A|260
          A|261     A|264     A|307     A|308     A|309     A|325     A|326
          A|327     A|346      B|23      B|27      B|34      B|37      B|39
          B|147     B|148     B|149     B|150     B|151     B|158     B|159
          B|168     B|169     B|170     B|171     B|172     B|173     B|174
          B|175     B|186     B|187     B|188     B|216     B|217     B|220
          B|229     B|259     B|260     B|261     B|264     B|307     B|308
          B|309     B|325     B|326     B|327     B|346
  
 Residues pruned out by function/max condition:
          A|149!    A|169!    A|170!    A|171!    A|172!    A|173!    A|187!
          A|188!    A|346!   A|ENDA     B|149!    B|169!    B|170!    B|171!
          B|172!    B|173!    B|187!    B|188!    B|346!   B|ENDA 
  
 Total number of residues for which LSSR restraints have been pruned out is       77 out of      698 participating
 Number of LSSR restraints pruned out is      4600 this is   13.6% of the total           33845
  
 -autoncs .Gelly cards suitable for adaptation for manual definition/pruning
           (note auto pruning is done in a slightly different way so exact results may differ slightly)
 -autoncs option .Gelly cards also written to file  auto_gelly_cards.txt
NOTE BUSTER_SET ncsautoXcld = Water    # card added - produced by -autoncs
NOTE BUSTER_SET ncsautoXcld = ncsautoXcld + { A|23 A|27 A|34 A|37 A|39 A|147 }    # card added - produced by -autoncs
NOTE BUSTER_SET ncsautoXcld = ncsautoXcld + { A|148 A|149 A|150 A|151 A|158 }    # card added - produced by -autoncs
NOTE BUSTER_SET ncsautoXcld = ncsautoXcld + { A|159 A|168 A|169 A|170 A|171 }    # card added - produced by -autoncs
NOTE BUSTER_SET ncsautoXcld = ncsautoXcld + { A|172 A|173 A|174 A|175 A|187 }    # card added - produced by -autoncs
NOTE BUSTER_SET ncsautoXcld = ncsautoXcld + { A|188 A|216 A|217 A|220 A|229 }    # card added - produced by -autoncs
NOTE BUSTER_SET ncsautoXcld = ncsautoXcld + { A|259 A|260 A|261 A|264 A|307 }    # card added - produced by -autoncs
NOTE BUSTER_SET ncsautoXcld = ncsautoXcld + { A|308 A|309 A|325 A|326 A|327 }    # card added - produced by -autoncs
NOTE BUSTER_SET ncsautoXcld = ncsautoXcld + { A|346 A|ENDA }    # card added - produced by -autoncs
NOTE BUSTER_SET ncsautoABset = Chain_A \ ncsautoXcld    # card added - produced by -autoncs
NOTE BUSTER_SIM_DEFINE ncsautoAB ncsautoABset B    # card added - produced by -autoncs
NOTE BUSTER_SIM_RESTRAIN_LSSR ncsautoAB 6.666    # card added - produced by -autoncs

 LSSR function contribution before pruning     32406.7
                             after pruning      6624.2
 Graph of which residues pruned from LSSR: ncs_pruned.mtv
 Graph of LSSR function outliers ncs_function_outliers.mtv
 Graph of LSSR gradient outliers ncs_gradient_outliers.mtv
 LSSR - analysis of distance difference distribution for NCS
         DeltaD less than 0.5 Angs:  proportion of population= 99.10% average DeltaD of subset     0.007 Angs
 Histogram of LSSR deltaD distribution ncs_histogram.mtv
 
 Interpreting card: NOTE BUSTER_TLS_SET tls1 { A|* }
 Interpreting card: NOTE BUSTER_TLS_SET tls2 { B|* }
 Interpreting card: NOTE BUSTER_TLS_SET tls1 { A|* }
 Interpreting card: NOTE BUSTER_TLS_T   tls1     -1.64775E-01 -3.36840E-02  2.15737E-01 -3.93502E-02 -3.35143E-02 -2.21740E-02
 Interpreting card: NOTE BUSTER_TLS_L   tls1      1.33783E+00  1.61817E+00  1.44183E+00 -3.13773E-01  8.90801E-01 -8.51731E-01
 Interpreting card: NOTE BUSTER_TLS_S   tls1      1.51047E-01 -2.84462E-01  3.13261E-01  6.85951E-02 -1.30648E-01 -2.56570E-01  7.59762E-02  1.23108E-01
 Interpreting card: NOTE BUSTER_TLS_O   tls1      2.31588E+01 -6.71772E+01  1.06797E+01
 Interpreting card: NOTE BUSTER_TLS_SET tls2 { B|* }
 Interpreting card: NOTE BUSTER_TLS_T   tls2     -2.34737E-01 -9.54492E-02  3.04843E-01  1.30665E-02 -3.15132E-02  3.78123E-02
 Interpreting card: NOTE BUSTER_TLS_L   tls2      1.51690E+00  1.05873E+00  1.03399E+00  6.62845E-01  9.72762E-01  7.91924E-01
 Interpreting card: NOTE BUSTER_TLS_S   tls2      8.67167E-04  4.13351E-02 -2.05843E-01 -1.01273E-01 -1.11597E-01  4.88749E-02  1.27152E-01 -6.61129E-02
 Interpreting card: NOTE BUSTER_TLS_O   tls2     -2.22253E+01 -7.89989E+01  3.21640E+00
 Interpreting card: NOTE BUSTER_TLS_FIX tls1 ALL
 Interpreting card: NOTE BUSTER_TLS_FIX tls2 ALL


 TLS information. Total number of groups:     2


 Group   Natoms    FixRB   FixTLS   Group Tag    Group Spec
     1     2768      Y        Y     tls1    { A|* }
     2     2758      Y        Y     tls2    { B|* }
 
NOTE BUSTER_TLS_SET tls1 { A|* }
NOTE BUSTER_TLS_T   tls1     -1.64775E-01 -3.36840E-02  2.15737E-01 -3.93502E-02 -3.35143E-02 -2.21740E-02
NOTE BUSTER_TLS_L   tls1      1.33783E+00  1.61817E+00  1.44183E+00 -3.13773E-01  8.90801E-01 -8.51731E-01
NOTE BUSTER_TLS_S   tls1      1.51047E-01 -2.84462E-01  3.13261E-01  6.85951E-02 -1.30648E-01 -2.56570E-01  7.59762E-02  1.23108E-01
NOTE BUSTER_TLS_O   tls1      2.31588E+01 -6.71772E+01  1.06797E+01
NOTE BUSTER_TLS_FIX tls1 ALL
 
NOTE BUSTER_TLS_SET tls2 { B|* }
NOTE BUSTER_TLS_T   tls2     -2.34737E-01 -9.54492E-02  3.04843E-01  1.30665E-02 -3.15132E-02  3.78123E-02
NOTE BUSTER_TLS_L   tls2      1.51690E+00  1.05873E+00  1.03399E+00  6.62845E-01  9.72762E-01  7.91924E-01
NOTE BUSTER_TLS_S   tls2      8.67167E-04  4.13351E-02 -2.05843E-01 -1.01273E-01 -1.11597E-01  4.88749E-02  1.27152E-01 -6.61129E-02
NOTE BUSTER_TLS_O   tls2     -2.22253E+01 -7.89989E+01  3.21640E+00
NOTE BUSTER_TLS_FIX tls2 ALL


 TLS: mmCIF group specification begin


#
loop_
_pdbx_refine_tls_group.id
_pdbx_refine_tls_group.refine_tls_id
_pdbx_refine_tls_group.beg_PDB_ins_code
_pdbx_refine_tls_group.beg_auth_asym_id
_pdbx_refine_tls_group.beg_auth_seq_id
_pdbx_refine_tls_group.end_PDB_ins_code
_pdbx_refine_tls_group.end_auth_asym_id
_pdbx_refine_tls_group.end_auth_seq_id
_pdbx_refine_tls_group.selection
_pdbx_refine_tls_group.selection_details
   1   1 . A   -1 . A  346 . '{ A|* }'
   2   1 . A  401 . A  401 . '{ A|* }'
   3   2 . B   -1 . B  346 . '{ B|* }'
#


 TLS: mmCIF group specification end


 Interpreting card: NOTE BUSTER_ANISO_SET Empty
 Interpreting card: NOTE BUSTER_ANISO_WEIGHT SCA  16.0
 Interpreting card: NOTE BUSTER_ANISO_WEIGHT SPH   0.6    0.6
 Interpreting card: NOTE BUSTER_ANISO_WEIGHT SIM   7.8    4.8    2.6    0
 Interpreting card: NOTE BUSTER_ANISO_WEIGHT RIG  21.5   21.5    3.7    0


 After TLS and ANISO: Number of atoms with fixed/combined xyz =     0
 After TLS and ANISO: Number of atoms with fixed/combined B   =     0
 After TLS and ANISO: Number of atoms with fixed/combined OCC =  5526
 After TLS and ANISO: Number of atoms with fixed/combined BIJ =  5526
 After TLS and ANISO: Number of atoms in a TLS group          =  5526
                                     cf total number of atoms =  5526
 

 ############### Setup state vector

 No free_vect_ulim_xyz argument found: using default of  100.000
 No free_vect_llim_xyz argument found: using default of -100.000
 Number of parameters added for TLS description                 =       0
 Number of parameters to describe molecular conformation        =   22104

 ############### Setup free vector

 Number of free parameters to be adjusted in refinement   =   22104

 ############### Setting up own geometry


 No command line argument '-tnt_fn' so will use gelly to do geometry function calculations

 ============= Call TNT functions


 ============= Get TNT symmetry information

 
      Getting symmetry operators from TNT. 
          gelly will classify symmetry using pdb-like convention: 
               SYMOP   SYMMETRY
              NNNMMM   OPERATOR
                1555   X,Y,Z
                2555   -Y,X-Y,Z
                3555   -X+Y,-X,Z
                4555   -X,-Y,1/2+Z
                5555   Y,-X+Y,1/2+Z
                6555   X-Y,X,1/2+Z
          where NNN -> operator number and MMM -> translation vector
 

 ============= Own Gelly geometry/restraints setup


 ----------- Identify bond-types


    Setting up GEOMBONDREC_TYPE_HASH* to be able to map a given bond type (e.g.
    "single") to an internal integer (GBOND_TYPE=1) etc.

    Using default  GEOMBONDREC_TYPE_HASH_STR='SING* 1 DOUB* 2 TRIP* 3 AROM* 4 DELO* 4 METAL* 5':
    to override default use a NOTE BUSTER_GEOMBONDREC_TYPE_HASH_STR gelly card.

 ----------- Classify torsion into peptide omega and other


 ----------- Setup of non-bonded terms

 Ideal-distance contact term setup:
    Restraint function constants (normally these values are from NOTE BUSTER_IDEAL_CONTACT_* cards)
    Sigma for general contacts      0.200 Angs
    Sigma for 1-4 contacts          0.100 Angs (contacts across a torsion)
    Sigma for 1-5 contacts          0.150 Angs
    Sigma for 1-N contacts          0.200 Angs
    Sigma for HBOND pairs           0.100 Angs (contacts between HB_types A and D or B's)
    Contact distance increment for each atom in HBOND          -0.150 Angs 
    Contact distance increment for H-bond acceptor              0.100 Angs 
    Contact distance increment for 1-4 with an O atom          -0.050 Angs 
    Contact distance increment for 1-4 with an N atom          -0.180 Angs 
    Contact distance increment for 1-4 with an C atom          -0.370 Angs 
    Contact distance increment for 1-4 with an S atom          -0.150 Angs 
    Contact distance increment for 1-4 with an P atom          -0.150 Angs 
    Contact distance increment for 1-4 with any other atom     -0.150 Angs 
    All atoms matched for CCP4 atom type.
    Number of CCP4 atom types used is         22 (H-bound and not H-bound counted separately)

 Setting up short/ideal-distance contacts
   Using gelly special position handling. (For TNT instead rerun with argument '-special_tnt')

   No command line argument '-special_all' specified. We will auto-generate
   EXCLUDE cards only for atom-atom contacts across symmetry (i.e. on special
   position) when this involves a contact within a residue to itself.

   Have set up non-bonded exclusions - 1-3 contacts
   Atom A|27:CA         makes the most 1-3 contacts:  6

   Have set up non-bonded exclusions - 1-4 contacts
   Atom A|27:N          makes the most 1-4 contacts:  7

   Have set up non-bonded exclusions - 1-5 contacts
   Atom A|28:C.A        makes the most 1-5 contacts: 10

 ############################################################################################################
 ###################################################### setting up contact terms
 ############################################################################################################


 ============= Call Gelly versions of TNT functions

 WARNING: 1-4 contact without 1-(2,3)-4 data for A|401:C02(LIG)       and A|401:C05(LIG)       X-X
 WARNING: 1-4 contact without 1-(2,3)-4 data for A|401:C03(LIG)       and A|401:C06(LIG)       X-X
 WARNING: 1-4 contact without 1-(2,3)-4 data for A|401:N04(LIG)       and A|401:N07(LIG)       X-X
 WARNING: 1-4 contact without 1-(2,3)-4 data for A|401:C06(LIG)       and A|401:C09(LIG)       X-X

       TERM      weight      number      TNT_fn    Gelly_fn        diff
       BOND       2.000        5642     613.378     613.378       0.000
       ANGL       2.000        7656    3423.426    3423.426       0.000
       IMPR       0.000           0       0.000       0.000       0.000
       TORS       2.000        1964    2651.053    2651.054      -0.000
       TRIG       2.000           0       0.000       0.000       0.000
       PLAN       5.000         958    1393.553    1393.553       0.000
       CONT       5.000           0      28.085       0.000      28.085
       BCOR      10.000        5632    1926.163    4357.585   -2431.423


 ============= Call Gelly geometry functions

   Switching torsion functional form to sinusoidal rather than split harmonic functional.
      if you want this not to happen then rerun with '-torsharm'
      Weight for sinusoidal torsion=     2.000
   BCOR function/gradient will be split between H and non-H atoms (as for any atom pair). Use '-bcorrel_all_in_hydrogens' to change this.

 ----------- Fix B-correlation restraints

 
 Checking for bonds that do not have a bcorrel restraint defined
 We will wipe all existing BCORrels if OCC=0.0 atom(s) is/are involved. This can be' switched off via the '-bcorrel_nowipe_zeroocc' flag.
 We will wipe all existing BCORrels if only non-hydrogen atom(s) with fixed B(iso) are involved. This can be switched off via the '-bcorrel_nowipe_fixb_nonhydrogens' flag.
 We will keep all existing BCORrels if hydrogen atom(s) with fixed B(iso) is/are involved. This can be switched off via the '-bcorrel_wipe_fixb_hydrogens' flag.
 No -bcorrel_missing_sigma found. So will set sigma to     2.5 Ang**2 for any bond with no existing BCORrel
 No -bcorrel_missing_sigma_hydrogens found. So will set sigma to     1.0 Ang**2 for any bond with no existing BCORrel involving hydrogens

 No active BCORels found involving OCC=0.0 atom(s)


 No active BCORels found involving fixed-B (only) non-hydrogen atom(s)

    add a default BCOR for bond A|401:C01(LIG) A|401:C02(LIG)
    add a default BCOR for bond A|401:C02(LIG) A|401:C03(LIG)
    add a default BCOR for bond A|401:C02(LIG) A|401:N07(LIG)
    add a default BCOR for bond A|401:C03(LIG) A|401:N04(LIG)
    add a default BCOR for bond A|401:N04(LIG) A|401:C05(LIG)
    add a default BCOR for bond A|401:C05(LIG) A|401:C06(LIG)
    add a default BCOR for bond A|401:C06(LIG) A|401:N07(LIG)
    add a default BCOR for bond A|401:N07(LIG) A|401:C08(LIG)
    add a default BCOR for bond A|401:C08(LIG) A|401:C09(LIG)
    add a default BCOR for bond A|401:C09(LIG) A|401:O10(LIG)

 Have added a total of        10 BCORrels for bonds which do not already have a BCORrel


 No bonds (for potential BCORrel addition) were skipped due to both atoms having constant/fixed B(iso)


 ----------- Deactivate hydrogen-free planes if hydrogen atoms present

 A total of       0 planes have been deactivated because they are duplicated hydrogen-free and with-hydrogen forms.

 ----------- Handle peptide omega angle(s)

      Torsion angles split into peptide omega and other
         Number of peptide omega angles is       698
         Number of other torsion is             1266
      Peptide omega angles are monitored rather than directly restrained (PLANE used instead).

 ----------- Fix cis-PRO

 Now apply the fix to get correct restraints for cis PRO residues
   Have got      11 bonds records and      26 angles
   from GEOMETRY cards for the special PROC residue
   Weight for ideal-distance contact term=     4.000

 ----------- Prepare SSBOND/LINK for output PDB file


 ----------- Prepare for QM


 ----------- Prepare symmetry in utility distance restraints


 ############### Setting up Rhofit contacts (if needed)

 No ligand-(fixed protein) contact term will be used

 ############### Working out initial function and gradient


   MaxLik Scaling Cycle 0002
 
 Initial functional value = 4.616232E+05 rms gradient=      75.3534
 X-ray contribution to function value = 4.374404E+05
 Geometry contribution (inc ncs)      =      24182.9
 Geometry function summary
     term                           number     weight       rms        GooF      function     fn/numb
     ================================================================================================
     BOND bond lengths (angs)       5642       2.000      0.0047       0.233       613.38       0.109
     ANGL bond angles (degs)        7656       2.000        0.79       0.473      3423.43       0.447
     TORS split-harmonic tors       1266       0.000       16.26       1.023         0.00       0.000
     SINTOR sinusoidal tors         1266       2.000       16.26                  2037.11       1.609
     PLAN general plane (angs)       958       5.000      0.0108       0.539      1393.55       1.455
     BCORrelations (angs^2)         5642      10.000       0.779       0.278      4363.33       0.773
     CONT (bad contacts)               0       5.000                                 0.00
     IDEAL(ideal-dist contact)      3788       4.000                              5698.75
     CHIRAL (gelly semiharmon)       742       5.000                                29.16
     SIM similarity restraint for NCS and/or TARGET                               6624.16
     OCCSUM restraint on sum of occupanies                                           0.00       as none defined
     DISTAN utility restraints interatomic distance                                  0.00       as none defined
     UTILANGLE utility angle restraints                                              0.00       as none defined
     UTILTOR utility restraints on torsion angles                                    0.00       as none defined
     Peptide omega torsion angles                           2.24 degs - monitor only

 Weighted rms      0.0047         0.793  (temporary rms bond and angle for BUSTER to pick up!)
 Weighted intra-residual rms(bond) for StdProtein (excluding H)      0.0044

 ############### Running initial check


 Initial Geometry Sanity Check

 Check for duplicate bonds: no duplicates found
 
 Check for duplicate or missing bond angles: all are OK
 Check for bond between an atom and itself not involving symmetry:  all are OK
   There are no bad contacts where Delta/sigma is greater than     6.5
   Worst contact dist=   2.632 std=   3.270 sigma=   0.200 abs(Delta/sigma)=    3.191 ats B|27:O (ILE) B|28:C.A (ASN)
   It is likely that the geometry libraries, sequence info and links match the input molecule
 GEOMETRY CHECK OVERALL:  PASS


 ############### Creating geometry/screen report


 ############### Option "tao" - run Tao optimizer

 TAO: Toolkit for Advanced Optimization. Reference: 
     Munson, T., Sarich, J., Wild, S., Benson, S., McInnes, L.C. (2018)
     "TAO User Manual (Revision 3.9)", Mathematics and Computer Science
     Division, Argonne National Laboratory
     ANL/MCS-TM-322, http://www.mcs.anl.gov/petsc/
 
 Have found -keeppdb flag so will write out a .pdb file every 1000000 its during opt
 Will apply TAO optimization 
 Maximum number of iterations      100
 Convergence test on Grms       4.0000 (-glim)
 Limit on rms displacement to original position (rmsD2init): NONE test inactive (-dlim)

    analysing argument no 0 : -autoncs with j=0
    analysing argument no 1 : -hydrogens_input_occ2zero with j=0
    analysing argument no 2 : -hydrogens_output_occ2heavy with j=0
    analysing argument no 3 : -screen with j=0
    analysing argument no 4 : 100 with j=0
    analysing argument no 5 : -screen_sigma with j=0
    analysing argument no 6 : 3.0 with j=0
    analysing argument no 7 : -keeppdb with j=0
    analysing argument no 8 : 1000000 with j=0
    analysing argument no 9 : -taomonitor_verbose with j=0
    found argument starting with "-tao" : -taomonitor_verbose
    analysing argument no 10 : 2 with j=1
    found value after -tao/-options_/-viewer_ argument : "2"
 PETSC/TAO: setting option "-taomonitor_verbose" = "2"


   MaxLik Scaling Cycle 0003
   Iters     Tot_Funct    Xray_Funct    Geom_Funct          Grms     rmsD2init     maxD2init             R         Rfree           LLG       LLGfree     rms(bond)    rms(angle)       Opttag_H
   Iters             K   B_IMPF_FRAG        K_MISS   B_TEMP_MISS   K_IMPF_MISS   B_IMPF_MISS        K_SOLV   B_TEMP_SOLV   K_IMPF_SOLV   B_IMPF_SOLV        B_TEMP     B_TEMP_11     B_TEMP_12     B_TEMP_22     B_TEMP_13     B_TEMP_23     B_TEMP_33      K_WILSON      B_WILSON      FGCYCLES       Scatag_H
   Iters          BOND         ANGLE      IMPROPER       TORSION    SINTORSION      TRIGONAL        PLANAR  BCORRELATION       CONTACT     IDEALDIST        CHIRAL    SIMILARITY       OCCSSUM  UTILDISTANCE     UTILANGLE   UTILTORSION        RHOFIT        ADP-01        ADP-02        ADP-03        ADP-04        ADP-05        ADP-06        ADP-07        ADP-08        ADP-09        ADP-10      RHOFITPC            QM       Funtag_H
       0  4.616216E+05  4.374403E+05       24181.3       75.3462  0.000000E+00  0.000000E+00      0.247406      0.244290  0.000000E+00  0.000000E+00    0.00468409      0.793120       Opttag_D
       0       60.1138       5.87176       1.00000  0.000000E+00       1.00000  0.000000E+00      0.924039       59.8959       1.00000       7.96481  0.000000E+00      0.302972  0.000000E+00      0.302972  0.000000E+00  0.000000E+00     -0.605943       57.3682      0.418910        2       Scatag_D
       0       613.378       3423.43  0.000000E+00  0.000000E+00       2037.11  0.000000E+00       1393.55       4361.75  0.000000E+00       5698.75       29.1571       6624.16 -1.000000E+10 -1.000000E+10 -1.000000E+10 -1.000000E+10 -1.000000E+10  0.000000E+00  0.000000E+00  0.000000E+00  0.000000E+00  0.000000E+00  0.000000E+00  0.000000E+00  0.000000E+00  0.000000E+00  0.000000E+00  0.000000E+00  0.000000E+00       Funtag_D
   Geometry Restraint Deviations SCREEN Information

 

 An atom has moved more than     1.50 A from the initial/last
 position - if this was an accepted trial point, probably need to update the
 non-bonded contact list.


   MaxLik Scaling Cycle 0004

   MaxLik Scaling Cycle 0005

   MaxLik Scaling Cycle 0006

   Wilson plot

   Statistics in resolution bins after refinement cycle 0001
```


|  |  |
| --- | --- |
|  | - Rxpct factors plot- Rxpct factors table- <Log-Lik Gain>- Ln Sigma A- Correlation Coefficients |


```
   Iters     Tot_Funct    Xray_Funct    Geom_Funct          Grms     rmsD2init     maxD2init             R         Rfree           LLG       LLGfree     rms(bond)    rms(angle)
   Iters             K   B_IMPF_FRAG        K_MISS   B_TEMP_MISS   K_IMPF_MISS   B_IMPF_MISS        K_SOLV   B_TEMP_SOLV   K_IMPF_SOLV   B_IMPF_SOLV        B_TEMP     B_TEMP_11     B_TEMP_12     B_TEMP_22     B_TEMP_13     B_TEMP_23     B_TEMP_33      K_WILSON      B_WILSON      FGCYCLES
   Iters          BOND         ANGLE      IMPROPER       TORSION    SINTORSION      TRIGONAL        PLANAR  BCORRELATION       CONTACT     IDEALDIST        CHIRAL    SIMILARITY       OCCSSUM  UTILDISTANCE     UTILANGLE   UTILTORSION        RHOFIT        ADP-01        ADP-02        ADP-03        ADP-04        ADP-05        ADP-06        ADP-07        ADP-08        ADP-09        ADP-10      RHOFITPC            QM
       1  4.605283E+05  4.366740E+05       23854.3       63.6336  9.252818E-04    0.00458315      0.246905      0.244131    0.00196212  8.858744E-04    0.00482609      0.794784       Opttag_D
       1       60.0717       5.79758       1.00000  0.000000E+00       1.00000  0.000000E+00      0.923267       59.7019       1.00000       8.10840  0.000000E+00      0.277977  0.000000E+00      0.277977  0.000000E+00  0.000000E+00     -0.555953       57.3682      0.418910        3       Scatag_D
       1       651.132       3437.81  0.000000E+00  0.000000E+00       2036.77  0.000000E+00       1404.30       3971.78  0.000000E+00       5697.88       29.6740       6625.01 -1.000000E+10 -1.000000E+10 -1.000000E+10 -1.000000E+10 -1.000000E+10  0.000000E+00  0.000000E+00  0.000000E+00  0.000000E+00  0.000000E+00  0.000000E+00  0.000000E+00  0.000000E+00  0.000000E+00  0.000000E+00  0.000000E+00  0.000000E+00       Funtag_D
   Geometry Restraint Deviations SCREEN Information

 

   MaxLik Scaling Cycle 0007

   Wilson plot

   Statistics in resolution bins after refinement cycle 0002
```


|  |  |
| --- | --- |
|  | - Rxpct factors plot- Rxpct factors table- <Log-Lik Gain>- Ln Sigma A- Correlation Coefficients |


```
   Iters     Tot_Funct    Xray_Funct    Geom_Funct          Grms     rmsD2init     maxD2init             R         Rfree           LLG       LLGfree     rms(bond)    rms(angle)
   Iters             K   B_IMPF_FRAG        K_MISS   B_TEMP_MISS   K_IMPF_MISS   B_IMPF_MISS        K_SOLV   B_TEMP_SOLV   K_IMPF_SOLV   B_IMPF_SOLV        B_TEMP     B_TEMP_11     B_TEMP_12     B_TEMP_22     B_TEMP_13     B_TEMP_23     B_TEMP_33      K_WILSON      B_WILSON      FGCYCLES
   Iters          BOND         ANGLE      IMPROPER       TORSION    SINTORSION      TRIGONAL        PLANAR  BCORRELATION       CONTACT     IDEALDIST        CHIRAL    SIMILARITY       OCCSSUM  UTILDISTANCE     UTILANGLE   UTILTORSION        RHOFIT        ADP-01        ADP-02        ADP-03        ADP-04        ADP-05        ADP-06        ADP-07        ADP-08        ADP-09        ADP-10      RHOFITPC            QM
       2  4.598219E+05  4.360165E+05       23805.4       49.9259    0.00194192    0.00978240      0.246402      0.243958    0.00367634    0.00164554    0.00504771      0.797856       Opttag_D
       2       60.0344       5.73549       1.00000  0.000000E+00       1.00000  0.000000E+00      0.922660       59.5714       1.00000       8.20735  0.000000E+00      0.254542  0.000000E+00      0.254542  0.000000E+00  0.000000E+00     -0.509084       57.3682      0.418910        1       Scatag_D
       2       712.307       3464.44  0.000000E+00  0.000000E+00       2036.57  0.000000E+00       1419.59       3806.97  0.000000E+00       5700.03       30.1541       6635.34 -1.000000E+10 -1.000000E+10 -1.000000E+10 -1.000000E+10 -1.000000E+10  0.000000E+00  0.000000E+00  0.000000E+00  0.000000E+00  0.000000E+00  0.000000E+00  0.000000E+00  0.000000E+00  0.000000E+00  0.000000E+00  0.000000E+00  0.000000E+00       Funtag_D
   Geometry Restraint Deviations SCREEN Information

 
       3  4.592937E+05  4.351468E+05       24146.9       54.5004    0.00343039     0.0173075      0.245726      0.243699    0.00594380    0.00270510    0.00539727      0.803824       Opttag_D
       3       59.9848       5.65503       1.00000  0.000000E+00       1.00000  0.000000E+00      0.921885       59.4170       1.00000       8.32703  0.000000E+00      0.224219  0.000000E+00      0.224219  0.000000E+00  0.000000E+00     -0.448437       57.3682      0.418910        1       Scatag_D
       3       814.378       3516.46  0.000000E+00  0.000000E+00       2036.20  0.000000E+00       1445.74       3931.41  0.000000E+00       5709.16       30.5656       6663.01 -1.000000E+10 -1.000000E+10 -1.000000E+10 -1.000000E+10 -1.000000E+10  0.000000E+00  0.000000E+00  0.000000E+00  0.000000E+00  0.000000E+00  0.000000E+00  0.000000E+00  0.000000E+00  0.000000E+00  0.000000E+00  0.000000E+00  0.000000E+00       Funtag_D
       4  4.588904E+05  4.342082E+05       24682.2       56.7728    0.00515459     0.0257947      0.244997      0.243409    0.00839111    0.00389173    0.00576205      0.808771       Opttag_D
       4       59.9313       5.57037       1.00000  0.000000E+00       1.00000  0.000000E+00      0.921083       59.2665       1.00000       8.44511  0.000000E+00      0.191789  0.000000E+00      0.191789  0.000000E+00  0.000000E+00     -0.383578       57.3682      0.418910        1       Scatag_D
       4       928.181       3559.87  0.000000E+00  0.000000E+00       2035.82  0.000000E+00       1481.07       4216.11  0.000000E+00       5722.65       30.5963       6707.90 -1.000000E+10 -1.000000E+10 -1.000000E+10 -1.000000E+10 -1.000000E+10  0.000000E+00  0.000000E+00  0.000000E+00  0.000000E+00  0.000000E+00  0.000000E+00  0.000000E+00  0.000000E+00  0.000000E+00  0.000000E+00  0.000000E+00  0.000000E+00       Funtag_D
       5  4.585224E+05  4.336994E+05       24822.9       45.9320    0.00622474     0.0306733      0.244596      0.243225    0.00971748    0.00463116    0.00589270      0.808478       Opttag_D
       5       59.9019       5.52564       1.00000  0.000000E+00       1.00000  0.000000E+00      0.920677       59.1988       1.00000       8.49947  0.000000E+00      0.174783  0.000000E+00      0.174783  0.000000E+00  0.000000E+00     -0.349566       57.3682      0.418910        1       Scatag_D
       5       970.750       3557.29  0.000000E+00  0.000000E+00       2035.65  0.000000E+00       1505.41       4255.35  0.000000E+00       5734.49       30.2382       6733.74 -1.000000E+10 -1.000000E+10 -1.000000E+10 -1.000000E+10 -1.000000E+10  0.000000E+00  0.000000E+00  0.000000E+00  0.000000E+00  0.000000E+00  0.000000E+00  0.000000E+00  0.000000E+00  0.000000E+00  0.000000E+00  0.000000E+00  0.000000E+00       Funtag_D
       6  4.578936E+05  4.324568E+05       25436.8       51.2947    0.00918031     0.0457661      0.243606      0.242735     0.0129574    0.00651110    0.00620486      0.819494       Opttag_D
       6       59.8337       5.42005       1.00000  0.000000E+00       1.00000  0.000000E+00      0.919754       59.0600       1.00000       8.61450  0.000000E+00      0.132666  0.000000E+00      0.132666  0.000000E+00  0.000000E+00     -0.265333       57.3682      0.418910        1       Scatag_D
       6       1076.32       3654.89  0.000000E+00  0.000000E+00       2035.19  0.000000E+00       1577.00       4481.17  0.000000E+00       5772.90       29.4532       6809.88 -1.000000E+10 -1.000000E+10 -1.000000E+10 -1.000000E+10 -1.000000E+10  0.000000E+00  0.000000E+00  0.000000E+00  0.000000E+00  0.000000E+00  0.000000E+00  0.000000E+00  0.000000E+00  0.000000E+00  0.000000E+00  0.000000E+00  0.000000E+00       Funtag_D
       7  4.575532E+05  4.312839E+05       26269.2       79.0590     0.0123168     0.0625114      0.242666      0.242246     0.0160154    0.00827209    0.00654407      0.844984       Opttag_D
       7       59.7765       5.32522       1.00000  0.000000E+00       1.00000  0.000000E+00      0.918960       58.9582       1.00000       8.70653  0.000000E+00     0.0907697  0.000000E+00     0.0907697  0.000000E+00  0.000000E+00     -0.181539       57.3682      0.418910        1       Scatag_D
       7       1197.22       3885.80  0.000000E+00  0.000000E+00       2035.00  0.000000E+00       1657.36       4752.29  0.000000E+00       5819.04       29.3593       6893.17 -1.000000E+10 -1.000000E+10 -1.000000E+10 -1.000000E+10 -1.000000E+10  0.000000E+00  0.000000E+00  0.000000E+00  0.000000E+00  0.000000E+00  0.000000E+00  0.000000E+00  0.000000E+00  0.000000E+00  0.000000E+00  0.000000E+00  0.000000E+00       Funtag_D
       8  4.571450E+05  4.309510E+05       26194.0       44.3129     0.0133243     0.0680582      0.242397      0.242094     0.0168833    0.00877719    0.00653540      0.832591       Opttag_D
       8       59.7627       5.29940       1.00000  0.000000E+00       1.00000  0.000000E+00      0.918748       58.9396       1.00000       8.72876  0.000000E+00     0.0784548  0.000000E+00     0.0784548  0.000000E+00  0.000000E+00     -0.156910       57.3682      0.418910        1       Scatag_D
       8       1194.05       3772.66  0.000000E+00  0.000000E+00       2034.86  0.000000E+00       1682.12       4736.65  0.000000E+00       5835.91       29.5345       6908.21 -1.000000E+10 -1.000000E+10 -1.000000E+10 -1.000000E+10 -1.000000E+10  0.000000E+00  0.000000E+00  0.000000E+00  0.000000E+00  0.000000E+00  0.000000E+00  0.000000E+00  0.000000E+00  0.000000E+00  0.000000E+00  0.000000E+00  0.000000E+00       Funtag_D
       9  4.567089E+05  4.302915E+05       26417.5       37.0262     0.0154479     0.0795599      0.241864      0.241787     0.0186029    0.00977169    0.00659392      0.836835       Opttag_D
       9       59.7378       5.24928       1.00000  0.000000E+00       1.00000  0.000000E+00      0.918329       58.9094       1.00000       8.77171  0.000000E+00     0.0536102  0.000000E+00     0.0536102  0.000000E+00  0.000000E+00     -0.107220       57.3682      0.418910        1       Scatag_D
       9       1215.53       3811.21  0.000000E+00  0.000000E+00       2034.68  0.000000E+00       1731.45       4789.28  0.000000E+00       5871.57       30.2351       6933.49 -1.000000E+10 -1.000000E+10 -1.000000E+10 -1.000000E+10 -1.000000E+10  0.000000E+00  0.000000E+00  0.000000E+00  0.000000E+00  0.000000E+00  0.000000E+00  0.000000E+00  0.000000E+00  0.000000E+00  0.000000E+00  0.000000E+00  0.000000E+00       Funtag_D
      10  4.563859E+05  4.296615E+05       26724.4       42.9286     0.0176108     0.0910471      0.241354      0.241501     0.0202453     0.0107091    0.00666114      0.844274       Opttag_D
      10       59.7152       5.20208       1.00000  0.000000E+00       1.00000  0.000000E+00      0.917920       58.8857       1.00000       8.81262  0.000000E+00     0.0301128  0.000000E+00     0.0301128  0.000000E+00  0.000000E+00    -0.0602257       57.3682      0.418910        1       Scatag_D
      10       1240.44       3879.28  0.000000E+00  0.000000E+00       2034.63  0.000000E+00       1778.66       4894.44  0.000000E+00       5909.16       31.0799       6956.71 -1.000000E+10 -1.000000E+10 -1.000000E+10 -1.000000E+10 -1.000000E+10  0.000000E+00  0.000000E+00  0.000000E+00  0.000000E+00  0.000000E+00  0.000000E+00  0.000000E+00  0.000000E+00  0.000000E+00  0.000000E+00  0.000000E+00  0.000000E+00       Funtag_D
      11  4.561434E+05  4.279528E+05       28190.6       110.887     0.0243491      0.129563      0.239972      0.240687     0.0247005     0.0131660    0.00697122      0.902338       Opttag_D
      11       59.6594       5.07843       1.00000  0.000000E+00       1.00000  0.000000E+00      0.916843       58.8592       1.00000       8.90717  0.000000E+00    -0.0334498  0.000000E+00    -0.0334498  0.000000E+00  0.000000E+00     0.0668997       57.3682      0.418910        1       Scatag_D
      11       1358.62       4431.21  0.000000E+00  0.000000E+00       2035.20  0.000000E+00       1918.41       5340.66  0.000000E+00       6034.80       33.8200       7037.93 -1.000000E+10 -1.000000E+10 -1.000000E+10 -1.000000E+10 -1.000000E+10  0.000000E+00  0.000000E+00  0.000000E+00  0.000000E+00  0.000000E+00  0.000000E+00  0.000000E+00  0.000000E+00  0.000000E+00  0.000000E+00  0.000000E+00  0.000000E+00       Funtag_D
      12  4.555058E+05  4.277368E+05       27769.0       39.7571     0.0254145      0.136021      0.239799      0.240566     0.0252635     0.0135082    0.00684684      0.864166       Opttag_D
      12       59.6528       5.06303       1.00000  0.000000E+00       1.00000  0.000000E+00      0.916680       58.8645       1.00000       8.91742  0.000000E+00    -0.0404514  0.000000E+00    -0.0404514  0.000000E+00  0.000000E+00     0.0809027       57.3682      0.418910        1       Scatag_D
      12       1310.57       4064.23  0.000000E+00  0.000000E+00       2035.31  0.000000E+00       1932.82       5298.82  0.000000E+00       6056.49       34.1189       7036.63 -1.000000E+10 -1.000000E+10 -1.000000E+10 -1.000000E+10 -1.000000E+10  0.000000E+00  0.000000E+00  0.000000E+00  0.000000E+00  0.000000E+00  0.000000E+00  0.000000E+00  0.000000E+00  0.000000E+00  0.000000E+00  0.000000E+00  0.000000E+00       Funtag_D
      13  4.552568E+05  4.276532E+05       27603.6       31.5665     0.0260390      0.139960      0.239729      0.240492     0.0254815     0.0136898    0.00679684      0.863728       Opttag_D
      13       59.6493       5.05664       1.00000  0.000000E+00       1.00000  0.000000E+00      0.916575       58.8741       1.00000       8.91989  0.000000E+00    -0.0415006  0.000000E+00    -0.0415006  0.000000E+00  0.000000E+00     0.0830012       57.3682      0.418910        1       Scatag_D
      13       1291.50       4060.11  0.000000E+00  0.000000E+00       2035.43  0.000000E+00       1931.61       5160.49  0.000000E+00       6067.75       33.8332       7022.88 -1.000000E+10 -1.000000E+10 -1.000000E+10 -1.000000E+10 -1.000000E+10  0.000000E+00  0.000000E+00  0.000000E+00  0.000000E+00  0.000000E+00  0.000000E+00  0.000000E+00  0.000000E+00  0.000000E+00  0.000000E+00  0.000000E+00  0.000000E+00       Funtag_D
      14  4.548982E+05  4.271849E+05       27713.3       40.2403     0.0287300      0.155785      0.239345      0.240197     0.0267025     0.0144479    0.00677049      0.871255       Opttag_D
      14       59.6343       5.02321       1.00000  0.000000E+00       1.00000  0.000000E+00      0.916187       58.8963       1.00000       8.93669  0.000000E+00    -0.0544176  0.000000E+00    -0.0544176  0.000000E+00  0.000000E+00      0.108835       57.3682      0.418910        1       Scatag_D
      14       1281.50       4131.18  0.000000E+00  0.000000E+00       2035.98  0.000000E+00       1956.66       5130.91  0.000000E+00       6121.60       33.7882       7021.72 -1.000000E+10 -1.000000E+10 -1.000000E+10 -1.000000E+10 -1.000000E+10  0.000000E+00  0.000000E+00  0.000000E+00  0.000000E+00  0.000000E+00  0.000000E+00  0.000000E+00  0.000000E+00  0.000000E+00  0.000000E+00  0.000000E+00  0.000000E+00       Funtag_D
      15  4.545938E+05  4.263918E+05       28202.0       75.5054     0.0333996      0.182259      0.238685      0.239701     0.0287703     0.0156526    0.00681530      0.896654       Opttag_D
      15       59.6123       4.96805       1.00000  0.000000E+00       1.00000  0.000000E+00      0.915551       58.9343       1.00000       8.96305  0.000000E+00    -0.0762917  0.000000E+00    -0.0762917  0.000000E+00  0.000000E+00      0.152583       57.3682      0.418910        1       Scatag_D
      15       1298.52       4375.55  0.000000E+00  0.000000E+00       2037.19  0.000000E+00       1999.92       5194.43  0.000000E+00       6219.94       33.9324       7042.53 -1.000000E+10 -1.000000E+10 -1.000000E+10 -1.000000E+10 -1.000000E+10  0.000000E+00  0.000000E+00  0.000000E+00  0.000000E+00  0.000000E+00  0.000000E+00  0.000000E+00  0.000000E+00  0.000000E+00  0.000000E+00  0.000000E+00  0.000000E+00       Funtag_D
      16  4.542051E+05  4.258287E+05       28376.4       38.6396     0.0367160      0.200639      0.238221      0.239375     0.0302385     0.0164020    0.00680082      0.888147       Opttag_D
      16       59.5995       4.93018       1.00000  0.000000E+00       1.00000  0.000000E+00      0.915138       58.9617       1.00000       8.98169  0.000000E+00    -0.0931871  0.000000E+00    -0.0931871  0.000000E+00  0.000000E+00      0.186374       57.3682      0.418910        1       Scatag_D
      16       1293.01       4292.92  0.000000E+00  0.000000E+00       2038.22  0.000000E+00       2038.41       5312.00  0.000000E+00       6295.94       34.3796       7071.56 -1.000000E+10 -1.000000E+10 -1.000000E+10 -1.000000E+10 -1.000000E+10  0.000000E+00  0.000000E+00  0.000000E+00  0.000000E+00  0.000000E+00  0.000000E+00  0.000000E+00  0.000000E+00  0.000000E+00  0.000000E+00  0.000000E+00  0.000000E+00       Funtag_D
      17  4.539123E+05  4.252941E+05       28618.2       29.3031     0.0397544      0.217000      0.237786      0.239080     0.0316322     0.0170584    0.00682942      0.893626       Opttag_D
      17       59.5891       4.89481       1.00000  0.000000E+00       1.00000  0.000000E+00      0.914740       58.9837       1.00000       9.00162  0.000000E+00     -0.109001  0.000000E+00     -0.109001  0.000000E+00  0.000000E+00      0.218002       57.3682      0.418910        1       Scatag_D
      17       1303.91       4346.05  0.000000E+00  0.000000E+00       2039.15  0.000000E+00       2069.46       5356.40  0.000000E+00       6367.29       34.9514       7100.96 -1.000000E+10 -1.000000E+10 -1.000000E+10 -1.000000E+10 -1.000000E+10  0.000000E+00  0.000000E+00  0.000000E+00  0.000000E+00  0.000000E+00  0.000000E+00  0.000000E+00  0.000000E+00  0.000000E+00  0.000000E+00  0.000000E+00  0.000000E+00       Funtag_D
      18  4.536349E+05  4.246344E+05       29000.6       39.3338     0.0436169      0.237196      0.237239      0.238736     0.0333524     0.0178160    0.00688744      0.904899       Opttag_D
      18       59.5791       4.85207       1.00000  0.000000E+00       1.00000  0.000000E+00      0.914234       59.0163       1.00000       9.02577  0.000000E+00     -0.127932  0.000000E+00     -0.127932  0.000000E+00  0.000000E+00      0.255864       57.3682      0.418910        1       Scatag_D
      18       1326.16       4456.39  0.000000E+00  0.000000E+00       2040.44  0.000000E+00       2103.24       5440.50  0.000000E+00       6459.81       35.9402       7138.07 -1.000000E+10 -1.000000E+10 -1.000000E+10 -1.000000E+10 -1.000000E+10  0.000000E+00  0.000000E+00  0.000000E+00  0.000000E+00  0.000000E+00  0.000000E+00  0.000000E+00  0.000000E+00  0.000000E+00  0.000000E+00  0.000000E+00  0.000000E+00       Funtag_D
      19  4.533879E+05  4.236781E+05       29709.8       55.1719     0.0496656      0.267851      0.236447      0.238267     0.0358455     0.0187956    0.00700904      0.922704       Opttag_D
      19       59.5698       4.79194       1.00000  0.000000E+00       1.00000  0.000000E+00      0.913477       59.0781       1.00000       9.05882  0.000000E+00     -0.154420  0.000000E+00     -0.154420  0.000000E+00  0.000000E+00      0.308839       57.3682      0.418910        1       Scatag_D
      19       1373.40       4633.49  0.000000E+00  0.000000E+00       2042.88  0.000000E+00       2154.21       5654.89  0.000000E+00       6610.85       38.0033       7202.08 -1.000000E+10 -1.000000E+10 -1.000000E+10 -1.000000E+10 -1.000000E+10  0.000000E+00  0.000000E+00  0.000000E+00  0.000000E+00  0.000000E+00  0.000000E+00  0.000000E+00  0.000000E+00  0.000000E+00  0.000000E+00  0.000000E+00  0.000000E+00       Funtag_D
      20  4.531177E+05  4.234843E+05       29633.4       30.4573     0.0507747      0.272528      0.236275      0.238189     0.0363509     0.0189934    0.00700226      0.916386       Opttag_D
      20       59.5693       4.77969       1.00000  0.000000E+00       1.00000  0.000000E+00      0.913290       59.0859       1.00000       9.06887  0.000000E+00     -0.158178  0.000000E+00     -0.158178  0.000000E+00  0.000000E+00      0.316356       57.3682      0.418910        1       Scatag_D
      20       1370.74       4570.26  0.000000E+00  0.000000E+00       2043.24  0.000000E+00       2154.34       5615.75  0.000000E+00       6635.76       38.3705       7204.96 -1.000000E+10 -1.000000E+10 -1.000000E+10 -1.000000E+10 -1.000000E+10  0.000000E+00  0.000000E+00  0.000000E+00  0.000000E+00  0.000000E+00  0.000000E+00  0.000000E+00  0.000000E+00  0.000000E+00  0.000000E+00  0.000000E+00  0.000000E+00       Funtag_D
      21  4.528547E+05  4.232211E+05       29633.6       29.8328     0.0523560      0.278587      0.236037      0.238089     0.0370372     0.0192667    0.00702967      0.917929       Opttag_D
      21       59.5698       4.76308       1.00000  0.000000E+00       1.00000  0.000000E+00      0.913011       59.0971       1.00000       9.08364  0.000000E+00     -0.162148  0.000000E+00     -0.162148  0.000000E+00  0.000000E+00      0.324297       57.3682      0.418910        1       Scatag_D
      21       1381.49       4585.65  0.000000E+00  0.000000E+00       2043.94  0.000000E+00       2152.39       5550.36  0.000000E+00       6671.96       38.5554       7209.26 -1.000000E+10 -1.000000E+10 -1.000000E+10 -1.000000E+10 -1.000000E+10  0.000000E+00  0.000000E+00  0.000000E+00  0.000000E+00  0.000000E+00  0.000000E+00  0.000000E+00  0.000000E+00  0.000000E+00  0.000000E+00  0.000000E+00  0.000000E+00       Funtag_D
      22  4.526084E+05  4.228037E+05       29804.7       34.4014     0.0551814      0.290080      0.235666      0.237903     0.0381254     0.0196839    0.00708642      0.922497       Opttag_D
      22       59.5719       4.73724       1.00000  0.000000E+00       1.00000  0.000000E+00      0.912591       59.1211       1.00000       9.10471  0.000000E+00     -0.169078  0.000000E+00     -0.169078  0.000000E+00  0.000000E+00      0.338156       57.3682      0.418910        1       Scatag_D
      22       1403.89       4631.41  0.000000E+00  0.000000E+00       2045.43  0.000000E+00       2161.76       5551.96  0.000000E+00       6741.99       38.7178       7229.55 -1.000000E+10 -1.000000E+10 -1.000000E+10 -1.000000E+10 -1.000000E+10  0.000000E+00  0.000000E+00  0.000000E+00  0.000000E+00  0.000000E+00  0.000000E+00  0.000000E+00  0.000000E+00  0.000000E+00  0.000000E+00  0.000000E+00  0.000000E+00       Funtag_D
      23  4.525195E+05  4.218703E+05       30649.2       79.6335     0.0623553      0.318422      0.234828      0.237443     0.0405591     0.0205443    0.00731429      0.952679       Opttag_D
      23       59.5868       4.68172       1.00000  0.000000E+00       1.00000  0.000000E+00      0.911652       59.1931       1.00000       9.15045  0.000000E+00     -0.183981  0.000000E+00     -0.183981  0.000000E+00  0.000000E+00      0.367962       57.3682      0.418910        1       Scatag_D
      23       1495.62       4939.42  0.000000E+00  0.000000E+00       2050.87  0.000000E+00       2197.30       5694.31  0.000000E+00       6928.74       39.1398       7303.81 -1.000000E+10 -1.000000E+10 -1.000000E+10 -1.000000E+10 -1.000000E+10  0.000000E+00  0.000000E+00  0.000000E+00  0.000000E+00  0.000000E+00  0.000000E+00  0.000000E+00  0.000000E+00  0.000000E+00  0.000000E+00  0.000000E+00  0.000000E+00       Funtag_D
      24  4.521508E+05  4.218625E+05       30288.3       25.7612     0.0624967      0.318767      0.234822      0.237423     0.0405793     0.0205608    0.00718429      0.929992       Opttag_D
      24       59.5888       4.68144       1.00000  0.000000E+00       1.00000  0.000000E+00      0.911636       59.1958       1.00000       9.15128  0.000000E+00     -0.183819  0.000000E+00     -0.183819  0.000000E+00  0.000000E+00      0.367639       57.3682      0.418910        1       Scatag_D
      24       1442.93       4706.98  0.000000E+00  0.000000E+00       2050.73  0.000000E+00       2198.44       5625.91  0.000000E+00       6932.28       39.0714       7291.97 -1.000000E+10 -1.000000E+10 -1.000000E+10 -1.000000E+10 -1.000000E+10  0.000000E+00  0.000000E+00  0.000000E+00  0.000000E+00  0.000000E+00  0.000000E+00  0.000000E+00  0.000000E+00  0.000000E+00  0.000000E+00  0.000000E+00  0.000000E+00       Funtag_D
      25  4.520353E+05  4.218147E+05       30220.6       19.9684     0.0630097      0.320395      0.234781      0.237373     0.0407039     0.0206266    0.00714811      0.929415       Opttag_D
      25       59.5931       4.67910       1.00000  0.000000E+00       1.00000  0.000000E+00      0.911583       59.2035       1.00000       9.15403  0.000000E+00     -0.184359  0.000000E+00     -0.184359  0.000000E+00  0.000000E+00      0.368717       57.3682      0.418910        1       Scatag_D
      25       1428.44       4701.13  0.000000E+00  0.000000E+00       2051.10  0.000000E+00       2201.55       5567.05  0.000000E+00       6945.36       39.1030       7286.87 -1.000000E+10 -1.000000E+10 -1.000000E+10 -1.000000E+10 -1.000000E+10  0.000000E+00  0.000000E+00  0.000000E+00  0.000000E+00  0.000000E+00  0.000000E+00  0.000000E+00  0.000000E+00  0.000000E+00  0.000000E+00  0.000000E+00  0.000000E+00       Funtag_D
      26  4.518509E+05  4.215602E+05       30290.7       26.6581     0.0655549      0.328988      0.234552      0.237195     0.0413674     0.0208728    0.00710943      0.932753       Opttag_D
      26       59.6102       4.66619       1.00000  0.000000E+00       1.00000  0.000000E+00      0.911313       59.2419       1.00000       9.16617  0.000000E+00     -0.187955  0.000000E+00     -0.187955  0.000000E+00  0.000000E+00      0.375910       57.3682      0.418910        1       Scatag_D
      26       1413.02       4734.96  0.000000E+00  0.000000E+00       2053.30  0.000000E+00       2215.81       5529.86  0.000000E+00       7011.58       39.5143       7292.65 -1.000000E+10 -1.000000E+10 -1.000000E+10 -1.000000E+10 -1.000000E+10  0.000000E+00  0.000000E+00  0.000000E+00  0.000000E+00  0.000000E+00  0.000000E+00  0.000000E+00  0.000000E+00  0.000000E+00  0.000000E+00  0.000000E+00  0.000000E+00       Funtag_D
      27  4.516665E+05  4.211720E+05       30494.5       48.3205     0.0699856      0.342610      0.234181      0.236910     0.0423797     0.0212086    0.00707560      0.943317       Opttag_D
      27       59.6493       4.64923       1.00000  0.000000E+00       1.00000  0.000000E+00      0.910901       59.3226       1.00000       9.18304  0.000000E+00     -0.193801  0.000000E+00     -0.193801  0.000000E+00  0.000000E+00      0.387601       57.3682      0.418910        1       Scatag_D
      27       1399.60       4842.82  0.000000E+00  0.000000E+00       2057.07  0.000000E+00       2240.95       5478.76  0.000000E+00       7127.25       40.4343       7307.64 -1.000000E+10 -1.000000E+10 -1.000000E+10 -1.000000E+10 -1.000000E+10  0.000000E+00  0.000000E+00  0.000000E+00  0.000000E+00  0.000000E+00  0.000000E+00  0.000000E+00  0.000000E+00  0.000000E+00  0.000000E+00  0.000000E+00  0.000000E+00       Funtag_D
      28  4.514546E+05  4.208612E+05       30593.3       27.1931     0.0737201      0.353889      0.233881      0.236707     0.0431899     0.0214173    0.00702045      0.940588       Opttag_D
      28       59.6832       4.63626       1.00000  0.000000E+00       1.00000  0.000000E+00      0.910573       59.3899       1.00000       9.19770  0.000000E+00     -0.198871  0.000000E+00     -0.198871  0.000000E+00  0.000000E+00      0.397742       57.3682      0.418910        1       Scatag_D
      28       1377.87       4814.85  0.000000E+00  0.000000E+00       2060.99  0.000000E+00       2263.79       5482.60  0.000000E+00       7225.00       41.1812       7327.05 -1.000000E+10 -1.000000E+10 -1.000000E+10 -1.000000E+10 -1.000000E+10  0.000000E+00  0.000000E+00  0.000000E+00  0.000000E+00  0.000000E+00  0.000000E+00  0.000000E+00  0.000000E+00  0.000000E+00  0.000000E+00  0.000000E+00  0.000000E+00       Funtag_D
      29  4.513068E+05  4.206697E+05       30637.1       20.5875     0.0760595      0.363388      0.233695      0.236595     0.0436893     0.0215524    0.00700342      0.942209       Opttag_D
      29       59.7104       4.62961       1.00000  0.000000E+00       1.00000  0.000000E+00      0.910382       59.4348       1.00000       9.20828  0.000000E+00     -0.203075  0.000000E+00     -0.203075  0.000000E+00  0.000000E+00      0.406151       57.3682      0.418910        1       Scatag_D
      29       1371.19       4831.45  0.000000E+00  0.000000E+00       2063.57  0.000000E+00       2276.76       5432.34  0.000000E+00       7283.36       41.6135       7336.84 -1.000000E+10 -1.000000E+10 -1.000000E+10 -1.000000E+10 -1.000000E+10  0.000000E+00  0.000000E+00  0.000000E+00  0.000000E+00  0.000000E+00  0.000000E+00  0.000000E+00  0.000000E+00  0.000000E+00  0.000000E+00  0.000000E+00  0.000000E+00       Funtag_D
      30  4.511382E+05  4.203704E+05       30767.8       26.3902     0.0796465      0.385740      0.233408      0.236431     0.0444696     0.0217728    0.00700537      0.946959       Opttag_D
      30       59.7576       4.62011       1.00000  0.000000E+00       1.00000  0.000000E+00      0.910103       59.5022       1.00000       9.22846  0.000000E+00     -0.211042  0.000000E+00     -0.211042  0.000000E+00  0.000000E+00      0.422084       57.3682      0.418910        1       Scatag_D
      30       1371.96       4880.29  0.000000E+00  0.000000E+00       2067.31  0.000000E+00       2296.79       5386.61  0.000000E+00       7371.17       42.2410       7351.41 -1.000000E+10 -1.000000E+10 -1.000000E+10 -1.000000E+10 -1.000000E+10  0.000000E+00  0.000000E+00  0.000000E+00  0.000000E+00  0.000000E+00  0.000000E+00  0.000000E+00  0.000000E+00  0.000000E+00  0.000000E+00  0.000000E+00  0.000000E+00       Funtag_D
      31  4.510176E+05  4.199698E+05       31047.8       37.6038     0.0843014      0.415185      0.233039      0.236204     0.0455141     0.0220867    0.00704802      0.954220       Opttag_D
      31       59.8238       4.60790       1.00000  0.000000E+00       1.00000  0.000000E+00      0.909757       59.5855       1.00000       9.26007  0.000000E+00     -0.223834  0.000000E+00     -0.223834  0.000000E+00  0.000000E+00      0.447668       57.3682      0.418910        1       Scatag_D
      31       1388.72       4955.42  0.000000E+00  0.000000E+00       2072.38  0.000000E+00       2324.87       5404.04  0.000000E+00       7484.66       42.8877       7374.87 -1.000000E+10 -1.000000E+10 -1.000000E+10 -1.000000E+10 -1.000000E+10  0.000000E+00  0.000000E+00  0.000000E+00  0.000000E+00  0.000000E+00  0.000000E+00  0.000000E+00  0.000000E+00  0.000000E+00  0.000000E+00  0.000000E+00  0.000000E+00       Funtag_D
      32  4.508728E+05  4.198667E+05       31006.1       20.5940     0.0850078      0.420713      0.232955      0.236149     0.0457829     0.0222199    0.00704198      0.950202       Opttag_D
      32       59.8396       4.60452       1.00000  0.000000E+00       1.00000  0.000000E+00      0.909706       59.5930       1.00000       9.27100  0.000000E+00     -0.228857  0.000000E+00     -0.228857  0.000000E+00  0.000000E+00      0.457714       57.3682      0.418910        1       Scatag_D
      32       1386.33       4913.77  0.000000E+00  0.000000E+00       2072.92  0.000000E+00       2328.30       5390.71  0.000000E+00       7496.94       42.8943       7374.26 -1.000000E+10 -1.000000E+10 -1.000000E+10 -1.000000E+10 -1.000000E+10  0.000000E+00  0.000000E+00  0.000000E+00  0.000000E+00  0.000000E+00  0.000000E+00  0.000000E+00  0.000000E+00  0.000000E+00  0.000000E+00  0.000000E+00  0.000000E+00       Funtag_D
      33  4.507165E+05  4.196433E+05       31073.3       20.2619     0.0865275      0.432625      0.232773      0.236019     0.0463654     0.0225138    0.00708485      0.950651       Opttag_D
      33       59.8730       4.59705       1.00000  0.000000E+00       1.00000  0.000000E+00      0.909603       59.6087       1.00000       9.29388  0.000000E+00     -0.239938  0.000000E+00     -0.239938  0.000000E+00  0.000000E+00      0.479876       57.3682      0.418910        1       Scatag_D
      33       1403.27       4918.42  0.000000E+00  0.000000E+00       2074.15  0.000000E+00       2337.24       5395.14  0.000000E+00       7524.00       42.9167       7378.13 -1.000000E+10 -1.000000E+10 -1.000000E+10 -1.000000E+10 -1.000000E+10  0.000000E+00  0.000000E+00  0.000000E+00  0.000000E+00  0.000000E+00  0.000000E+00  0.000000E+00  0.000000E+00  0.000000E+00  0.000000E+00  0.000000E+00  0.000000E+00       Funtag_D
      34  4.505868E+05  4.193742E+05       31212.6       23.0363     0.0886798      0.448888      0.232548      0.235857     0.0470669     0.0228251    0.00713865      0.952187       Opttag_D
      34       59.9181       4.58897       1.00000  0.000000E+00       1.00000  0.000000E+00      0.909488       59.6381       1.00000       9.31945  0.000000E+00     -0.253226  0.000000E+00     -0.253226  0.000000E+00  0.000000E+00      0.506452       57.3682      0.418910        1       Scatag_D
      34       1424.66       4934.32  0.000000E+00  0.000000E+00       2076.46  0.000000E+00       2351.70       5428.16  0.000000E+00       7566.09       42.9977       7388.23 -1.000000E+10 -1.000000E+10 -1.000000E+10 -1.000000E+10 -1.000000E+10  0.000000E+00  0.000000E+00  0.000000E+00  0.000000E+00  0.000000E+00  0.000000E+00  0.000000E+00  0.000000E+00  0.000000E+00  0.000000E+00  0.000000E+00  0.000000E+00       Funtag_D
      35  4.504975E+05  4.187255E+05       31772.0       53.7207     0.0944041      0.492626      0.231984      0.235471     0.0487582     0.0235032    0.00732644      0.965575       Opttag_D
      35       60.0456       4.57295       1.00000  0.000000E+00       1.00000  0.000000E+00      0.909289       59.7258       1.00000       9.38132  0.000000E+00     -0.287818  0.000000E+00     -0.287818  0.000000E+00  0.000000E+00      0.575636       57.3682      0.418910        1       Scatag_D
      35       1500.60       5074.06  0.000000E+00  0.000000E+00       2082.65  0.000000E+00       2394.47       5575.30  0.000000E+00       7679.35       43.1671       7422.37 -1.000000E+10 -1.000000E+10 -1.000000E+10 -1.000000E+10 -1.000000E+10  0.000000E+00  0.000000E+00  0.000000E+00  0.000000E+00  0.000000E+00  0.000000E+00  0.000000E+00  0.000000E+00  0.000000E+00  0.000000E+00  0.000000E+00  0.000000E+00       Funtag_D
      36  4.502840E+05  4.186476E+05       31636.3       19.2249     0.0951868      0.499237      0.231910      0.235423     0.0489613     0.0235790    0.00726565      0.955366       Opttag_D
      36       60.0653       4.57193       1.00000  0.000000E+00       1.00000  0.000000E+00      0.909289       59.7413       1.00000       9.38722  0.000000E+00     -0.292380  0.000000E+00     -0.292380  0.000000E+00  0.000000E+00      0.584761       57.3682      0.418910        1       Scatag_D
      36       1475.80       4967.33  0.000000E+00  0.000000E+00       2083.98  0.000000E+00       2399.20       5553.22  0.000000E+00       7693.18       43.0047       7420.64 -1.000000E+10 -1.000000E+10 -1.000000E+10 -1.000000E+10 -1.000000E+10  0.000000E+00  0.000000E+00  0.000000E+00  0.000000E+00  0.000000E+00  0.000000E+00  0.000000E+00  0.000000E+00  0.000000E+00  0.000000E+00  0.000000E+00  0.000000E+00       Funtag_D
      37  4.502003E+05  4.186078E+05       31592.6       15.4352     0.0955996      0.503655      0.231867      0.235392     0.0490652     0.0236339    0.00724265      0.954840       Opttag_D
      37       60.0790       4.57211       1.00000  0.000000E+00       1.00000  0.000000E+00      0.909314       59.7518       1.00000       9.38871  0.000000E+00     -0.295005  0.000000E+00     -0.295005  0.000000E+00  0.000000E+00      0.590010       57.3682      0.418910        1       Scatag_D
      37       1466.47       4961.87  0.000000E+00  0.000000E+00       2084.71  0.000000E+00       2399.68       5523.65  0.000000E+00       7697.85       42.8756       7415.47 -1.000000E+10 -1.000000E+10 -1.000000E+10 -1.000000E+10 -1.000000E+10  0.000000E+00  0.000000E+00  0.000000E+00  0.000000E+00  0.000000E+00  0.000000E+00  0.000000E+00  0.000000E+00  0.000000E+00  0.000000E+00  0.000000E+00  0.000000E+00       Funtag_D
      38  4.500820E+05  4.184326E+05       31649.4       20.0481     0.0974735      0.520777      0.231696      0.235269     0.0495220     0.0238359    0.00722490      0.955818       Opttag_D
      38       60.1324       4.57146       1.00000  0.000000E+00       1.00000  0.000000E+00      0.909358       59.7914       1.00000       9.40169  0.000000E+00     -0.306189  0.000000E+00     -0.306189  0.000000E+00  0.000000E+00      0.612378       57.3682      0.418910        1       Scatag_D
      38       1459.29       4972.03  0.000000E+00  0.000000E+00       2087.26  0.000000E+00       2407.79       5541.04  0.000000E+00       7728.21       42.7894       7411.01 -1.000000E+10 -1.000000E+10 -1.000000E+10 -1.000000E+10 -1.000000E+10  0.000000E+00  0.000000E+00  0.000000E+00  0.000000E+00  0.000000E+00  0.000000E+00  0.000000E+00  0.000000E+00  0.000000E+00  0.000000E+00  0.000000E+00  0.000000E+00       Funtag_D
      39  4.499771E+05  4.181346E+05       31842.5       39.2169      0.101010      0.553320      0.231415      0.235019     0.0502988     0.0241935    0.00721647      0.959959       Opttag_D
      39       60.2349       4.57252       1.00000  0.000000E+00       1.00000  0.000000E+00      0.909468       59.8652       1.00000       9.42571  0.000000E+00     -0.325916  0.000000E+00     -0.325916  0.000000E+00  0.000000E+00      0.651832       57.3682      0.418910        1       Scatag_D
      39       1455.89       5015.20  0.000000E+00  0.000000E+00       2092.15  0.000000E+00       2419.99       5628.36  0.000000E+00       7786.16       42.8599       7401.85 -1.000000E+10 -1.000000E+10 -1.000000E+10 -1.000000E+10 -1.000000E+10  0.000000E+00  0.000000E+00  0.000000E+00  0.000000E+00  0.000000E+00  0.000000E+00  0.000000E+00  0.000000E+00  0.000000E+00  0.000000E+00  0.000000E+00  0.000000E+00       Funtag_D
      40  4.498398E+05  4.179677E+05       31872.1       19.6758      0.103205      0.572843      0.231260      0.234874     0.0507340     0.0243745    0.00719280      0.957906       Opttag_D
      40       60.2975       4.57406       1.00000  0.000000E+00       1.00000  0.000000E+00      0.909540       59.9117       1.00000       9.44028  0.000000E+00     -0.337908  0.000000E+00     -0.337908  0.000000E+00  0.000000E+00      0.675816       57.3682      0.418910        1       Scatag_D
      40       1446.35       4993.78  0.000000E+00  0.000000E+00       2095.09  0.000000E+00       2428.67       5642.79  0.000000E+00       7823.84       42.9906       7398.57 -1.000000E+10 -1.000000E+10 -1.000000E+10 -1.000000E+10 -1.000000E+10  0.000000E+00  0.000000E+00  0.000000E+00  0.000000E+00  0.000000E+00  0.000000E+00  0.000000E+00  0.000000E+00  0.000000E+00  0.000000E+00  0.000000E+00  0.000000E+00       Funtag_D
      41  4.497463E+05  4.178379E+05       31908.4       14.5104      0.104979      0.589276      0.231142      0.234757     0.0510724     0.0245235    0.00717902      0.958219       Opttag_D
      41       60.3499       4.57602       1.00000  0.000000E+00       1.00000  0.000000E+00      0.909611       59.9485       1.00000       9.45167  0.000000E+00     -0.347340  0.000000E+00     -0.347340  0.000000E+00  0.000000E+00      0.694680       57.3682      0.418910        1       Scatag_D
      41       1440.82       4997.05  0.000000E+00  0.000000E+00       2097.52  0.000000E+00       2432.47       5653.29  0.000000E+00       7852.37       43.1781       7391.71 -1.000000E+10 -1.000000E+10 -1.000000E+10 -1.000000E+10 -1.000000E+10  0.000000E+00  0.000000E+00  0.000000E+00  0.000000E+00  0.000000E+00  0.000000E+00  0.000000E+00  0.000000E+00  0.000000E+00  0.000000E+00  0.000000E+00  0.000000E+00       Funtag_D
      42  4.496524E+05  4.176683E+05       31984.1       18.3730      0.107322      0.611694      0.230986      0.234631     0.0515146     0.0247193    0.00717614      0.959556       Opttag_D
      42       60.4214       4.57918       1.00000  0.000000E+00       1.00000  0.000000E+00      0.909729       59.9957       1.00000       9.46596  0.000000E+00     -0.359633  0.000000E+00     -0.359633  0.000000E+00  0.000000E+00      0.719266       57.3682      0.418910        1       Scatag_D
      42       1439.66       5011.00  0.000000E+00  0.000000E+00       2100.87  0.000000E+00       2435.44       5685.22  0.000000E+00       7887.34       43.3634       7381.22 -1.000000E+10 -1.000000E+10 -1.000000E+10 -1.000000E+10 -1.000000E+10  0.000000E+00  0.000000E+00  0.000000E+00  0.000000E+00  0.000000E+00  0.000000E+00  0.000000E+00  0.000000E+00  0.000000E+00  0.000000E+00  0.000000E+00  0.000000E+00       Funtag_D
      43  4.495803E+05  4.173865E+05       32193.8       35.2006      0.111261      0.650075      0.230739      0.234459     0.0522494     0.0249615    0.00719572      0.963446       Opttag_D
      43       60.5437       4.58501       1.00000  0.000000E+00       1.00000  0.000000E+00      0.909953       60.0700       1.00000       9.49022  0.000000E+00     -0.379933  0.000000E+00     -0.379933  0.000000E+00  0.000000E+00      0.759866       57.3682      0.418910        1       Scatag_D
      43       1447.53       5051.71  0.000000E+00  0.000000E+00       2107.28  0.000000E+00       2439.87       5789.61  0.000000E+00       7943.89       43.4250       7370.49 -1.000000E+10 -1.000000E+10 -1.000000E+10 -1.000000E+10 -1.000000E+10  0.000000E+00  0.000000E+00  0.000000E+00  0.000000E+00  0.000000E+00  0.000000E+00  0.000000E+00  0.000000E+00  0.000000E+00  0.000000E+00  0.000000E+00  0.000000E+00       Funtag_D
      44  4.494693E+05  4.173350E+05       32134.3       16.3094      0.111724      0.656033      0.230693      0.234430     0.0523836     0.0250179    0.00719476      0.960523       Opttag_D
      44       60.5613       4.58519       1.00000  0.000000E+00       1.00000  0.000000E+00      0.909996       60.0739       1.00000       9.49325  0.000000E+00     -0.382490  0.000000E+00     -0.382490  0.000000E+00  0.000000E+00      0.764980       57.3682      0.418910        1       Scatag_D
      44       1447.14       5021.10  0.000000E+00  0.000000E+00       2108.02  0.000000E+00       2437.39       5765.80  0.000000E+00       7944.93       43.2543       7366.64 -1.000000E+10 -1.000000E+10 -1.000000E+10 -1.000000E+10 -1.000000E+10  0.000000E+00  0.000000E+00  0.000000E+00  0.000000E+00  0.000000E+00  0.000000E+00  0.000000E+00  0.000000E+00  0.000000E+00  0.000000E+00  0.000000E+00  0.000000E+00       Funtag_D
      45  4.493925E+05  4.172813E+05       32111.2       14.1755      0.112002      0.661354      0.230644      0.234406     0.0525236     0.0250644    0.00720867      0.959606       Opttag_D
      45       60.5753       4.58458       1.00000  0.000000E+00       1.00000  0.000000E+00      0.910040       60.0704       1.00000       9.49510  0.000000E+00     -0.384264  0.000000E+00     -0.384264  0.000000E+00  0.000000E+00      0.768528       57.3682      0.418910        1       Scatag_D
      45       1452.74       5011.52  0.000000E+00  0.000000E+00       2108.71  0.000000E+00       2433.13       5760.97  0.000000E+00       7939.67       42.9122       7361.53 -1.000000E+10 -1.000000E+10 -1.000000E+10 -1.000000E+10 -1.000000E+10  0.000000E+00  0.000000E+00  0.000000E+00  0.000000E+00  0.000000E+00  0.000000E+00  0.000000E+00  0.000000E+00  0.000000E+00  0.000000E+00  0.000000E+00  0.000000E+00       Funtag_D
      46  4.493152E+05  4.171770E+05       32138.2       16.5403      0.112995      0.674565      0.230547      0.234353     0.0527956     0.0251429    0.00722805      0.959173       Opttag_D
      46       60.6130       4.58518       1.00000  0.000000E+00       1.00000  0.000000E+00      0.910140       60.0765       1.00000       9.50102  0.000000E+00     -0.389020  0.000000E+00     -0.389020  0.000000E+00  0.000000E+00      0.778039       57.3682      0.418910        1       Scatag_D
      46       1460.57       5006.99  0.000000E+00  0.000000E+00       2110.75  0.000000E+00       2429.18       5788.51  0.000000E+00       7942.29       42.5888       7357.35 -1.000000E+10 -1.000000E+10 -1.000000E+10 -1.000000E+10 -1.000000E+10  0.000000E+00  0.000000E+00  0.000000E+00  0.000000E+00  0.000000E+00  0.000000E+00  0.000000E+00  0.000000E+00  0.000000E+00  0.000000E+00  0.000000E+00  0.000000E+00       Funtag_D
      47  4.492458E+05  4.170099E+05       32235.9       37.2348      0.114947      0.702223      0.230389      0.234254     0.0532314     0.0252798    0.00725391      0.960622       Opttag_D
      47       60.6896       4.58887       1.00000  0.000000E+00       1.00000  0.000000E+00      0.910373       60.0874       1.00000       9.50864  0.000000E+00     -0.395710  0.000000E+00     -0.395710  0.000000E+00  0.000000E+00      0.791421       57.3682      0.418910        1       Scatag_D
      47       1471.03       5022.14  0.000000E+00  0.000000E+00       2115.21  0.000000E+00       2420.70       5868.96  0.000000E+00       7943.44       42.2138       7352.21 -1.000000E+10 -1.000000E+10 -1.000000E+10 -1.000000E+10 -1.000000E+10  0.000000E+00  0.000000E+00  0.000000E+00  0.000000E+00  0.000000E+00  0.000000E+00  0.000000E+00  0.000000E+00  0.000000E+00  0.000000E+00  0.000000E+00  0.000000E+00       Funtag_D
      48  4.491323E+05  4.169410E+05       32191.3       16.4149      0.116036      0.716135      0.230322      0.234204     0.0534108     0.0253420    0.00722969      0.957193       Opttag_D
      48       60.7290       4.59170       1.00000  0.000000E+00       1.00000  0.000000E+00      0.910491       60.0974       1.00000       9.51292  0.000000E+00     -0.398925  0.000000E+00     -0.398925  0.000000E+00  0.000000E+00      0.797849       57.3682      0.418910        1       Scatag_D
      48       1461.23       4986.35  0.000000E+00  0.000000E+00       2117.67  0.000000E+00       2418.76       5863.17  0.000000E+00       7949.92       42.0886       7352.11 -1.000000E+10 -1.000000E+10 -1.000000E+10 -1.000000E+10 -1.000000E+10  0.000000E+00  0.000000E+00  0.000000E+00  0.000000E+00  0.000000E+00  0.000000E+00  0.000000E+00  0.000000E+00  0.000000E+00  0.000000E+00  0.000000E+00  0.000000E+00       Funtag_D
      49  4.490706E+05  4.169201E+05       32150.5       12.8986      0.116647      0.725007      0.230298      0.234166     0.0534654     0.0253857    0.00719713      0.956485       Opttag_D
      49       60.7525       4.59453       1.00000  0.000000E+00       1.00000  0.000000E+00      0.910574       60.1036       1.00000       9.51329  0.000000E+00     -0.399506  0.000000E+00     -0.399506  0.000000E+00  0.000000E+00      0.799012       57.3682      0.418910        1       Scatag_D
      49       1448.10       4978.98  0.000000E+00  0.000000E+00       2119.21  0.000000E+00       2416.55       5847.16  0.000000E+00       7951.52       42.1013       7346.92 -1.000000E+10 -1.000000E+10 -1.000000E+10 -1.000000E+10 -1.000000E+10  0.000000E+00  0.000000E+00  0.000000E+00  0.000000E+00  0.000000E+00  0.000000E+00  0.000000E+00  0.000000E+00  0.000000E+00  0.000000E+00  0.000000E+00  0.000000E+00       Funtag_D
      50  4.489966E+05  4.168850E+05       32111.6       15.7558      0.117754      0.742192      0.230253      0.234094     0.0535568     0.0254678    0.00714763      0.955809       Opttag_D
      50       60.7975       4.60030       1.00000  0.000000E+00       1.00000  0.000000E+00      0.910749       60.1136       1.00000       9.51293  0.000000E+00     -0.400411  0.000000E+00     -0.400411  0.000000E+00  0.000000E+00      0.800822       57.3682      0.418910        1       Scatag_D
      50       1428.25       4971.93  0.000000E+00  0.000000E+00       2122.17  0.000000E+00       2412.18       5846.47  0.000000E+00       7950.95       42.0467       7337.62 -1.000000E+10 -1.000000E+10 -1.000000E+10 -1.000000E+10 -1.000000E+10  0.000000E+00  0.000000E+00  0.000000E+00  0.000000E+00  0.000000E+00  0.000000E+00  0.000000E+00  0.000000E+00  0.000000E+00  0.000000E+00  0.000000E+00  0.000000E+00       Funtag_D
      51  4.489422E+05  4.168200E+05       32122.2       31.2366      0.119677      0.772951      0.230178      0.233979     0.0537264     0.0255838    0.00710307      0.956447       Opttag_D
      51       60.8773       4.61059       1.00000  0.000000E+00       1.00000  0.000000E+00      0.911069       60.1292       1.00000       9.51289  0.000000E+00     -0.403049  0.000000E+00     -0.403049  0.000000E+00  0.000000E+00      0.806097       57.3682      0.418910        1       Scatag_D
      51       1410.49       4978.58  0.000000E+00  0.000000E+00       2127.42  0.000000E+00       2406.81       5884.88  0.000000E+00       7948.65       41.7574       7323.64 -1.000000E+10 -1.000000E+10 -1.000000E+10 -1.000000E+10 -1.000000E+10  0.000000E+00  0.000000E+00  0.000000E+00  0.000000E+00  0.000000E+00  0.000000E+00  0.000000E+00  0.000000E+00  0.000000E+00  0.000000E+00  0.000000E+00  0.000000E+00       Funtag_D
      52  4.488577E+05  4.167935E+05       32064.2       14.3251      0.120359      0.784568      0.230151      0.233931     0.0537954     0.0256221    0.00707155      0.954487       Opttag_D
      52       60.9065       4.61421       1.00000  0.000000E+00       1.00000  0.000000E+00      0.911190       60.1326       1.00000       9.51345  0.000000E+00     -0.404104  0.000000E+00     -0.404104  0.000000E+00  0.000000E+00      0.808207       57.3682      0.418910        1       Scatag_D
      52       1398.00       4958.19  0.000000E+00  0.000000E+00       2129.35  0.000000E+00       2405.16       5868.41  0.000000E+00       7945.71       41.6465       7317.71 -1.000000E+10 -1.000000E+10 -1.000000E+10 -1.000000E+10 -1.000000E+10  0.000000E+00  0.000000E+00  0.000000E+00  0.000000E+00  0.000000E+00  0.000000E+00  0.000000E+00  0.000000E+00  0.000000E+00  0.000000E+00  0.000000E+00  0.000000E+00       Funtag_D
      53  4.488030E+05  4.167552E+05       32047.8       11.5389      0.120897      0.795150      0.230116      0.233887     0.0538954     0.0256666    0.00706415      0.954277       Opttag_D
      53       60.9312       4.61638       1.00000  0.000000E+00       1.00000  0.000000E+00      0.911290       60.1311       1.00000       9.51549  0.000000E+00     -0.405747  0.000000E+00     -0.405747  0.000000E+00  0.000000E+00      0.811494       57.3682      0.418910        1       Scatag_D
      53       1395.08       4956.01  0.000000E+00  0.000000E+00       2130.98  0.000000E+00       2404.06       5869.65  0.000000E+00       7940.49       41.4839       7310.08 -1.000000E+10 -1.000000E+10 -1.000000E+10 -1.000000E+10 -1.000000E+10  0.000000E+00  0.000000E+00  0.000000E+00  0.000000E+00  0.000000E+00  0.000000E+00  0.000000E+00  0.000000E+00  0.000000E+00  0.000000E+00  0.000000E+00  0.000000E+00       Funtag_D
      54  4.487429E+05  4.166862E+05       32056.7       14.5188      0.121810      0.812936      0.230059      0.233812     0.0540752     0.0257323    0.00706347      0.954460       Opttag_D
      54       60.9717       4.61956       1.00000  0.000000E+00       1.00000  0.000000E+00      0.911445       60.1271       1.00000       9.52006  0.000000E+00     -0.408672  0.000000E+00     -0.408672  0.000000E+00  0.000000E+00      0.817345       57.3682      0.418910        1       Scatag_D
      54       1394.81       4957.91  0.000000E+00  0.000000E+00       2133.72  0.000000E+00       2403.15       5893.94  0.000000E+00       7933.41       41.2907       7298.43 -1.000000E+10 -1.000000E+10 -1.000000E+10 -1.000000E+10 -1.000000E+10  0.000000E+00  0.000000E+00  0.000000E+00  0.000000E+00  0.000000E+00  0.000000E+00  0.000000E+00  0.000000E+00  0.000000E+00  0.000000E+00  0.000000E+00  0.000000E+00       Funtag_D
      55  4.486648E+05  4.165402E+05       32124.6       26.9550      0.123565      0.848304      0.229943      0.233667     0.0544558     0.0258438    0.00708682      0.955533       Opttag_D
      55       61.0474       4.62432       1.00000  0.000000E+00       1.00000  0.000000E+00      0.911713       60.1099       1.00000       9.53291  0.000000E+00     -0.414100  0.000000E+00     -0.414100  0.000000E+00  0.000000E+00      0.828199       57.3682      0.418910        1       Scatag_D
      55       1404.05       4969.07  0.000000E+00  0.000000E+00       2139.17  0.000000E+00       2403.75       5970.91  0.000000E+00       7920.60       41.1479       7275.90 -1.000000E+10 -1.000000E+10 -1.000000E+10 -1.000000E+10 -1.000000E+10  0.000000E+00  0.000000E+00  0.000000E+00  0.000000E+00  0.000000E+00  0.000000E+00  0.000000E+00  0.000000E+00  0.000000E+00  0.000000E+00  0.000000E+00  0.000000E+00       Funtag_D
      56  4.485773E+05  4.164296E+05       32147.7       16.0493      0.125067      0.877707      0.229859      0.233551     0.0547443     0.0259192    0.00709844      0.954903       Opttag_D
      56       61.1097       4.62875       1.00000  0.000000E+00       1.00000  0.000000E+00      0.911931       60.0958       1.00000       9.54422  0.000000E+00     -0.418656  0.000000E+00     -0.418656  0.000000E+00  0.000000E+00      0.837313       57.3682      0.418910        1       Scatag_D
      56       1408.66       4962.52  0.000000E+00  0.000000E+00       2143.82  0.000000E+00       2405.78       6011.45  0.000000E+00       7912.66       41.0859       7261.73 -1.000000E+10 -1.000000E+10 -1.000000E+10 -1.000000E+10 -1.000000E+10  0.000000E+00  0.000000E+00  0.000000E+00  0.000000E+00  0.000000E+00  0.000000E+00  0.000000E+00  0.000000E+00  0.000000E+00  0.000000E+00  0.000000E+00  0.000000E+00       Funtag_D
      57  4.485185E+05  4.163791E+05       32139.4       12.2138      0.125452      0.887523      0.229822      0.233506     0.0548758     0.0259691    0.00710553      0.954600       Opttag_D
      57       61.1262       4.62853       1.00000  0.000000E+00       1.00000  0.000000E+00      0.911967       60.0829       1.00000       9.55082  0.000000E+00     -0.420300  0.000000E+00     -0.420300  0.000000E+00  0.000000E+00      0.840600       57.3682      0.418910        1       Scatag_D
      57       1411.47       4959.37  0.000000E+00  0.000000E+00       2145.23  0.000000E+00       2407.03       6011.77  0.000000E+00       7908.56       41.1576       7254.79 -1.000000E+10 -1.000000E+10 -1.000000E+10 -1.000000E+10 -1.000000E+10  0.000000E+00  0.000000E+00  0.000000E+00  0.000000E+00  0.000000E+00  0.000000E+00  0.000000E+00  0.000000E+00  0.000000E+00  0.000000E+00  0.000000E+00  0.000000E+00       Funtag_D
      58  4.484473E+05  4.162882E+05       32159.1       15.6963      0.126183      0.907092      0.229763      0.233425     0.0551129     0.0260680    0.00712222      0.954766       Opttag_D
      58       61.1582       4.62851       1.00000  0.000000E+00       1.00000  0.000000E+00      0.912037       60.0563       1.00000       9.56409  0.000000E+00     -0.423426  0.000000E+00     -0.423426  0.000000E+00  0.000000E+00      0.846852       57.3682      0.418910        1       Scatag_D
      58       1418.11       4961.09  0.000000E+00  0.000000E+00       2148.04  0.000000E+00       2409.84       6037.40  0.000000E+00       7900.24       41.3218       7243.09 -1.000000E+10 -1.000000E+10 -1.000000E+10 -1.000000E+10 -1.000000E+10  0.000000E+00  0.000000E+00  0.000000E+00  0.000000E+00  0.000000E+00  0.000000E+00  0.000000E+00  0.000000E+00  0.000000E+00  0.000000E+00  0.000000E+00  0.000000E+00       Funtag_D
      59  4.484034E+05  4.161947E+05       32208.7       20.4901      0.127237      0.933179      0.229700      0.233333     0.0553567     0.0261901    0.00713590      0.955218       Opttag_D
      59       61.2036       4.63065       1.00000  0.000000E+00       1.00000  0.000000E+00      0.912163       60.0268       1.00000       9.57905  0.000000E+00     -0.427467  0.000000E+00     -0.427467  0.000000E+00  0.000000E+00      0.854933       57.3682      0.418910        1       Scatag_D
      59       1423.56       4965.79  0.000000E+00  0.000000E+00       2151.89  0.000000E+00       2413.49       6086.97  0.000000E+00       7890.67       41.4321       7234.90 -1.000000E+10 -1.000000E+10 -1.000000E+10 -1.000000E+10 -1.000000E+10  0.000000E+00  0.000000E+00  0.000000E+00  0.000000E+00  0.000000E+00  0.000000E+00  0.000000E+00  0.000000E+00  0.000000E+00  0.000000E+00  0.000000E+00  0.000000E+00       Funtag_D
      60  4.483530E+05  4.161889E+05       32164.0       11.6611      0.127299      0.936575      0.229698      0.233330     0.0553717     0.0262176    0.00712339      0.954620       Opttag_D
      60       61.2080       4.63104       1.00000  0.000000E+00       1.00000  0.000000E+00      0.912172       60.0216       1.00000       9.58089  0.000000E+00     -0.427959  0.000000E+00     -0.427959  0.000000E+00  0.000000E+00      0.855919       57.3682      0.418910        1       Scatag_D
      60       1418.57       4959.58  0.000000E+00  0.000000E+00       2152.27  0.000000E+00       2413.34       6059.88  0.000000E+00       7888.38       41.4139       7230.58 -1.000000E+10 -1.000000E+10 -1.000000E+10 -1.000000E+10 -1.000000E+10  0.000000E+00  0.000000E+00  0.000000E+00  0.000000E+00  0.000000E+00  0.000000E+00  0.000000E+00  0.000000E+00  0.000000E+00  0.000000E+00  0.000000E+00  0.000000E+00       Funtag_D
      61  4.482947E+05  4.161624E+05       32132.3       10.7675      0.127804      0.951237      0.229681      0.233298     0.0554408     0.0263040    0.00710469      0.954381       Opttag_D
      61       61.2320       4.63359       1.00000  0.000000E+00       1.00000  0.000000E+00      0.912244       60.0070       1.00000       9.58731  0.000000E+00     -0.429983  0.000000E+00     -0.429983  0.000000E+00  0.000000E+00      0.859966       57.3682      0.418910        1       Scatag_D
      61       1411.14       4957.09  0.000000E+00  0.000000E+00       2154.23  0.000000E+00       2413.59       6050.74  0.000000E+00       7882.43       41.4155       7221.65 -1.000000E+10 -1.000000E+10 -1.000000E+10 -1.000000E+10 -1.000000E+10  0.000000E+00  0.000000E+00  0.000000E+00  0.000000E+00  0.000000E+00  0.000000E+00  0.000000E+00  0.000000E+00  0.000000E+00  0.000000E+00  0.000000E+00  0.000000E+00       Funtag_D
      62  4.482543E+05  4.161329E+05       32121.4       11.6991      0.128382      0.967575      0.229663      0.233257     0.0555179     0.0263917    0.00709150      0.954161       Opttag_D
      62       61.2579       4.63626       1.00000  0.000000E+00       1.00000  0.000000E+00      0.912318       59.9915       1.00000       9.59405  0.000000E+00     -0.432048  0.000000E+00     -0.432048  0.000000E+00  0.000000E+00      0.864095       57.3682      0.418910        1       Scatag_D
      62       1405.90       4954.80  0.000000E+00  0.000000E+00       2156.39  0.000000E+00       2413.90       6056.72  0.000000E+00       7876.91       41.5055       7215.27 -1.000000E+10 -1.000000E+10 -1.000000E+10 -1.000000E+10 -1.000000E+10  0.000000E+00  0.000000E+00  0.000000E+00  0.000000E+00  0.000000E+00  0.000000E+00  0.000000E+00  0.000000E+00  0.000000E+00  0.000000E+00  0.000000E+00  0.000000E+00       Funtag_D
      63  4.482170E+05  4.160663E+05       32150.7       25.0063      0.129988       1.01317      0.229616      0.233150     0.0556914     0.0266398    0.00707755      0.954495       Opttag_D
      63       61.3266       4.64385       1.00000  0.000000E+00       1.00000  0.000000E+00      0.912509       59.9458       1.00000       9.61241  0.000000E+00     -0.436770  0.000000E+00     -0.436770  0.000000E+00  0.000000E+00      0.873540       57.3682      0.418910        1       Scatag_D
      63       1400.38       4958.28  0.000000E+00  0.000000E+00       2162.37  0.000000E+00       2414.31       6102.94  0.000000E+00       7864.02       41.8180       7206.58 -1.000000E+10 -1.000000E+10 -1.000000E+10 -1.000000E+10 -1.000000E+10  0.000000E+00  0.000000E+00  0.000000E+00  0.000000E+00  0.000000E+00  0.000000E+00  0.000000E+00  0.000000E+00  0.000000E+00  0.000000E+00  0.000000E+00  0.000000E+00       Funtag_D
      64  4.481529E+05  4.160548E+05       32098.1       9.62334      0.130206       1.02028      0.229603      0.233130     0.0557213     0.0266642    0.00706629      0.953602       Opttag_D
      64       61.3356       4.64466       1.00000  0.000000E+00       1.00000  0.000000E+00      0.912522       59.9400       1.00000       9.61476  0.000000E+00     -0.437499  0.000000E+00     -0.437499  0.000000E+00  0.000000E+00      0.874998       57.3682      0.418910        1       Scatag_D
      64       1395.92       4949.00  0.000000E+00  0.000000E+00       2163.20  0.000000E+00       2413.07       6069.25  0.000000E+00       7862.11       41.8268       7203.72 -1.000000E+10 -1.000000E+10 -1.000000E+10 -1.000000E+10 -1.000000E+10  0.000000E+00  0.000000E+00  0.000000E+00  0.000000E+00  0.000000E+00  0.000000E+00  0.000000E+00  0.000000E+00  0.000000E+00  0.000000E+00  0.000000E+00  0.000000E+00       Funtag_D
      65  4.481253E+05  4.160483E+05       32077.0       8.86675      0.130237       1.02317      0.229595      0.233122     0.0557384     0.0266810    0.00706486      0.953334       Opttag_D
      65       61.3369       4.64440       1.00000  0.000000E+00       1.00000  0.000000E+00      0.912509       59.9363       1.00000       9.61548  0.000000E+00     -0.437580  0.000000E+00     -0.437580  0.000000E+00  0.000000E+00      0.875160       57.3682      0.418910        1       Scatag_D
      65       1395.36       4946.22  0.000000E+00  0.000000E+00       2163.39  0.000000E+00       2411.06       6058.50  0.000000E+00       7861.23       41.8019       7199.41 -1.000000E+10 -1.000000E+10 -1.000000E+10 -1.000000E+10 -1.000000E+10  0.000000E+00  0.000000E+00  0.000000E+00  0.000000E+00  0.000000E+00  0.000000E+00  0.000000E+00  0.000000E+00  0.000000E+00  0.000000E+00  0.000000E+00  0.000000E+00       Funtag_D
      66  4.480790E+05  4.160173E+05       32061.7       11.1176      0.130687       1.04021      0.229568      0.233091     0.0558193     0.0267509    0.00706744      0.952945       Opttag_D
      66       61.3560       4.64555       1.00000  0.000000E+00       1.00000  0.000000E+00      0.912523       59.9170       1.00000       9.62198  0.000000E+00     -0.438878  0.000000E+00     -0.438878  0.000000E+00  0.000000E+00      0.877757       57.3682      0.418910        1       Scatag_D
      66       1396.38       4942.19  0.000000E+00  0.000000E+00       2165.26  0.000000E+00       2406.86       6061.28  0.000000E+00       7857.20       41.7483       7190.85 -1.000000E+10 -1.000000E+10 -1.000000E+10 -1.000000E+10 -1.000000E+10  0.000000E+00  0.000000E+00  0.000000E+00  0.000000E+00  0.000000E+00  0.000000E+00  0.000000E+00  0.000000E+00  0.000000E+00  0.000000E+00  0.000000E+00  0.000000E+00       Funtag_D
      67  4.480643E+05  4.159566E+05       32107.7       30.3384      0.131844       1.08005      0.229518      0.233047     0.0559776     0.0268456    0.00709139      0.953306       Opttag_D
      67       61.4050       4.64981       1.00000  0.000000E+00       1.00000  0.000000E+00      0.912589       59.8757       1.00000       9.63694  0.000000E+00     -0.441967  0.000000E+00     -0.441967  0.000000E+00  0.000000E+00      0.883935       57.3682      0.418910        1       Scatag_D
      67       1405.86       4945.93  0.000000E+00  0.000000E+00       2169.95  0.000000E+00       2399.60       6117.00  0.000000E+00       7849.69       41.5611       7178.14 -1.000000E+10 -1.000000E+10 -1.000000E+10 -1.000000E+10 -1.000000E+10  0.000000E+00  0.000000E+00  0.000000E+00  0.000000E+00  0.000000E+00  0.000000E+00  0.000000E+00  0.000000E+00  0.000000E+00  0.000000E+00  0.000000E+00  0.000000E+00       Funtag_D
      68  4.479972E+05  4.159462E+05       32051.1       11.6062      0.132064       1.08679      0.229510      0.233040     0.0560047     0.0268638    0.00708503      0.952533       Opttag_D
      68       61.4138       4.65065       1.00000  0.000000E+00       1.00000  0.000000E+00      0.912604       59.8681       1.00000       9.64014  0.000000E+00     -0.442424  0.000000E+00     -0.442424  0.000000E+00  0.000000E+00      0.884847       57.3682      0.418910        1       Scatag_D
      68       1403.34       4937.91  0.000000E+00  0.000000E+00       2170.77  0.000000E+00       2398.98       6073.11  0.000000E+00       7848.65       41.5645       7176.74 -1.000000E+10 -1.000000E+10 -1.000000E+10 -1.000000E+10 -1.000000E+10  0.000000E+00  0.000000E+00  0.000000E+00  0.000000E+00  0.000000E+00  0.000000E+00  0.000000E+00  0.000000E+00  0.000000E+00  0.000000E+00  0.000000E+00  0.000000E+00       Funtag_D
      69  4.479674E+05  4.159309E+05       32036.5       7.87208      0.132468       1.09922      0.229500      0.233034     0.0560445     0.0268802    0.00708457      0.952369       Opttag_D
      69       61.4307       4.65252       1.00000  0.000000E+00       1.00000  0.000000E+00      0.912634       59.8561       1.00000       9.64517  0.000000E+00     -0.443209  0.000000E+00     -0.443209  0.000000E+00  0.000000E+00      0.886419       57.3682      0.418910        1       Scatag_D
      69       1403.15       4936.22  0.000000E+00  0.000000E+00       2172.31  0.000000E+00       2397.66       6065.30  0.000000E+00       7846.65       41.5579       7173.67 -1.000000E+10 -1.000000E+10 -1.000000E+10 -1.000000E+10 -1.000000E+10  0.000000E+00  0.000000E+00  0.000000E+00  0.000000E+00  0.000000E+00  0.000000E+00  0.000000E+00  0.000000E+00  0.000000E+00  0.000000E+00  0.000000E+00  0.000000E+00       Funtag_D
      70  4.479326E+05  4.159085E+05       32024.2       9.68557      0.133267       1.12409      0.229488      0.233027     0.0561030     0.0268980    0.00708646      0.952311       Opttag_D
      70       61.4636       4.65661       1.00000  0.000000E+00       1.00000  0.000000E+00      0.912692       59.8338       1.00000       9.65417  0.000000E+00     -0.444407  0.000000E+00     -0.444407  0.000000E+00  0.000000E+00      0.888815       57.3682      0.418910        1       Scatag_D
      70       1403.90       4935.61  0.000000E+00  0.000000E+00       2175.32  0.000000E+00       2394.86       6062.52  0.000000E+00       7843.30       41.5899       7167.07 -1.000000E+10 -1.000000E+10 -1.000000E+10 -1.000000E+10 -1.000000E+10  0.000000E+00  0.000000E+00  0.000000E+00  0.000000E+00  0.000000E+00  0.000000E+00  0.000000E+00  0.000000E+00  0.000000E+00  0.000000E+00  0.000000E+00  0.000000E+00       Funtag_D
      71  4.478827E+05  4.158993E+05       31983.4       11.7097      0.134400       1.15929      0.229486      0.233025     0.0561270     0.0269033    0.00707526      0.952081       Opttag_D
      71       61.5092       4.66355       1.00000  0.000000E+00       1.00000  0.000000E+00      0.912768       59.8071       1.00000       9.66462  0.000000E+00     -0.444592  0.000000E+00     -0.444592  0.000000E+00  0.000000E+00      0.889184       57.3682      0.418910        1       Scatag_D
      71       1399.47       4933.23  0.000000E+00  0.000000E+00       2179.42  0.000000E+00       2391.38       6044.23  0.000000E+00       7839.48       41.8301       7154.39 -1.000000E+10 -1.000000E+10 -1.000000E+10 -1.000000E+10 -1.000000E+10  0.000000E+00  0.000000E+00  0.000000E+00  0.000000E+00  0.000000E+00  0.000000E+00  0.000000E+00  0.000000E+00  0.000000E+00  0.000000E+00  0.000000E+00  0.000000E+00       Funtag_D
      72  4.478512E+05  4.158970E+05       31954.2       13.6535      0.135533       1.19330      0.229489      0.233047     0.0561329     0.0268688    0.00706395      0.951922       Opttag_D
      72       61.5536       4.67070       1.00000  0.000000E+00       1.00000  0.000000E+00      0.912841       59.7831       1.00000       9.67472  0.000000E+00     -0.444627  0.000000E+00     -0.444627  0.000000E+00  0.000000E+00      0.889255       57.3682      0.418910        2       Scatag_D
      72       1395.00       4931.58  0.000000E+00  0.000000E+00       2183.39  0.000000E+00       2388.68       6033.96  0.000000E+00       7836.79       42.0784       7142.72 -1.000000E+10 -1.000000E+10 -1.000000E+10 -1.000000E+10 -1.000000E+10  0.000000E+00  0.000000E+00  0.000000E+00  0.000000E+00  0.000000E+00  0.000000E+00  0.000000E+00  0.000000E+00  0.000000E+00  0.000000E+00  0.000000E+00  0.000000E+00       Funtag_D
      73  4.478157E+05  4.159042E+05       31911.5       8.10831      0.136165       1.21254      0.229493      0.233072     0.0561141     0.0268503    0.00704910      0.951671       Opttag_D
      73       61.5784       4.67517       1.00000  0.000000E+00       1.00000  0.000000E+00      0.912872       59.7727       1.00000       9.67954  0.000000E+00     -0.443773  0.000000E+00     -0.443773  0.000000E+00  0.000000E+00      0.887546       57.3682      0.418910        1       Scatag_D
      73       1389.14       4928.98  0.000000E+00  0.000000E+00       2185.51  0.000000E+00       2387.40       6009.79  0.000000E+00       7835.50       42.3281       7132.90 -1.000000E+10 -1.000000E+10 -1.000000E+10 -1.000000E+10 -1.000000E+10  0.000000E+00  0.000000E+00  0.000000E+00  0.000000E+00  0.000000E+00  0.000000E+00  0.000000E+00  0.000000E+00  0.000000E+00  0.000000E+00  0.000000E+00  0.000000E+00       Funtag_D
      74  4.477832E+05  4.159077E+05       31875.6       9.40241      0.136592       1.22595      0.229498      0.233089     0.0561050     0.0268290    0.00703188      0.951473       Opttag_D
      74       61.5957       4.67817       1.00000  0.000000E+00       1.00000  0.000000E+00      0.912880       59.7676       1.00000       9.68295  0.000000E+00     -0.442700  0.000000E+00     -0.442700  0.000000E+00  0.000000E+00      0.885399       57.3682      0.418910        1       Scatag_D
      74       1382.36       4926.93  0.000000E+00  0.000000E+00       2186.86  0.000000E+00       2386.61       5993.85  0.000000E+00       7834.19       42.5459       7122.21 -1.000000E+10 -1.000000E+10 -1.000000E+10 -1.000000E+10 -1.000000E+10  0.000000E+00  0.000000E+00  0.000000E+00  0.000000E+00  0.000000E+00  0.000000E+00  0.000000E+00  0.000000E+00  0.000000E+00  0.000000E+00  0.000000E+00  0.000000E+00       Funtag_D
      75  4.477411E+05  4.159020E+05       31839.1       12.9650      0.137341       1.24845      0.229496      0.233117     0.0561197     0.0267890    0.00700935      0.951297       Opttag_D
      75       61.6259       4.68276       1.00000  0.000000E+00       1.00000  0.000000E+00      0.912885       59.7584       1.00000       9.69046  0.000000E+00     -0.441290  0.000000E+00     -0.441290  0.000000E+00  0.000000E+00      0.882580       57.3682      0.418910        1       Scatag_D
      75       1373.52       4925.10  0.000000E+00  0.000000E+00       2189.17  0.000000E+00       2385.85       5983.24  0.000000E+00       7832.52       42.8524       7106.83 -1.000000E+10 -1.000000E+10 -1.000000E+10 -1.000000E+10 -1.000000E+10  0.000000E+00  0.000000E+00  0.000000E+00  0.000000E+00  0.000000E+00  0.000000E+00  0.000000E+00  0.000000E+00  0.000000E+00  0.000000E+00  0.000000E+00  0.000000E+00       Funtag_D
      76  4.477014E+05  4.158798E+05       31821.5       15.3835      0.138095       1.26989      0.229486      0.233158     0.0561776     0.0267240    0.00699580      0.951424       Opttag_D
      76       61.6565       4.68634       1.00000  0.000000E+00       1.00000  0.000000E+00      0.912873       59.7490       1.00000       9.70054  0.000000E+00     -0.440108  0.000000E+00     -0.440108  0.000000E+00  0.000000E+00      0.880217       57.3682      0.418910        1       Scatag_D
      76       1368.21       4926.43  0.000000E+00  0.000000E+00       2191.47  0.000000E+00       2385.96       5978.95  0.000000E+00       7831.82       43.0243       7095.66 -1.000000E+10 -1.000000E+10 -1.000000E+10 -1.000000E+10 -1.000000E+10  0.000000E+00  0.000000E+00  0.000000E+00  0.000000E+00  0.000000E+00  0.000000E+00  0.000000E+00  0.000000E+00  0.000000E+00  0.000000E+00  0.000000E+00  0.000000E+00       Funtag_D
      77  4.476655E+05  4.158552E+05       31810.4       9.00181      0.138216       1.27279      0.229473      0.233165     0.0562419     0.0267024    0.00699778      0.951444       Opttag_D
      77       61.6620       4.68579       1.00000  0.000000E+00       1.00000  0.000000E+00      0.912856       59.7462       1.00000       9.70442  0.000000E+00     -0.440587  0.000000E+00     -0.440587  0.000000E+00  0.000000E+00      0.881175       57.3682      0.418910        1       Scatag_D
      77       1368.99       4926.63  0.000000E+00  0.000000E+00       2191.83  0.000000E+00       2385.88       5968.69  0.000000E+00       7831.69       42.9230       7093.73 -1.000000E+10 -1.000000E+10 -1.000000E+10 -1.000000E+10 -1.000000E+10  0.000000E+00  0.000000E+00  0.000000E+00  0.000000E+00  0.000000E+00  0.000000E+00  0.000000E+00  0.000000E+00  0.000000E+00  0.000000E+00  0.000000E+00  0.000000E+00       Funtag_D
      78  4.476329E+05  4.158091E+05       31823.8       8.09132      0.138430       1.27746      0.229447      0.233177     0.0563620     0.0266774    0.00700786      0.951548       Opttag_D
      78       61.6709       4.68450       1.00000  0.000000E+00       1.00000  0.000000E+00      0.912826       59.7394       1.00000       9.71195  0.000000E+00     -0.441597  0.000000E+00     -0.441597  0.000000E+00  0.000000E+00      0.883194       57.3682      0.418910        1       Scatag_D
      78       1372.93       4927.71  0.000000E+00  0.000000E+00       2192.49  0.000000E+00       2385.72       5978.40  0.000000E+00       7831.89       42.7321       7091.89 -1.000000E+10 -1.000000E+10 -1.000000E+10 -1.000000E+10 -1.000000E+10  0.000000E+00  0.000000E+00  0.000000E+00  0.000000E+00  0.000000E+00  0.000000E+00  0.000000E+00  0.000000E+00  0.000000E+00  0.000000E+00  0.000000E+00  0.000000E+00       Funtag_D
      79  4.476123E+05  4.157730E+05       31839.4       9.30722      0.138719       1.28423      0.229431      0.233194     0.0564562     0.0266325    0.00701604      0.951704       Opttag_D
      79       61.6820       4.68409       1.00000  0.000000E+00       1.00000  0.000000E+00      0.912801       59.7323       1.00000       9.71917  0.000000E+00     -0.442253  0.000000E+00     -0.442253  0.000000E+00  0.000000E+00      0.884507       57.3682      0.418910        1       Scatag_D
      79       1376.14       4929.32  0.000000E+00  0.000000E+00       2193.35  0.000000E+00       2386.07       5988.60  0.000000E+00       7832.57       42.6144       7090.70 -1.000000E+10 -1.000000E+10 -1.000000E+10 -1.000000E+10 -1.000000E+10  0.000000E+00  0.000000E+00  0.000000E+00  0.000000E+00  0.000000E+00  0.000000E+00  0.000000E+00  0.000000E+00  0.000000E+00  0.000000E+00  0.000000E+00  0.000000E+00       Funtag_D
      80  4.475831E+05  4.157138E+05       31869.3       8.18638      0.139301       1.29772      0.229403      0.233224     0.0566105     0.0265581    0.00702970      0.952088       Opttag_D
      80       61.7031       4.68385       1.00000  0.000000E+00       1.00000  0.000000E+00      0.912759       59.7189       1.00000       9.73260  0.000000E+00     -0.443298  0.000000E+00     -0.443298  0.000000E+00  0.000000E+00      0.886596       57.3682      0.418910        1       Scatag_D
      80       1381.50       4933.30  0.000000E+00  0.000000E+00       2195.01  0.000000E+00       2387.62       6004.69  0.000000E+00       7834.41       42.4621       7090.31 -1.000000E+10 -1.000000E+10 -1.000000E+10 -1.000000E+10 -1.000000E+10  0.000000E+00  0.000000E+00  0.000000E+00  0.000000E+00  0.000000E+00  0.000000E+00  0.000000E+00  0.000000E+00  0.000000E+00  0.000000E+00  0.000000E+00  0.000000E+00       Funtag_D
      81  4.475612E+05  4.156577E+05       31903.5       11.2752      0.139893       1.31111      0.229371      0.233236     0.0567568     0.0265138    0.00704052      0.952674       Opttag_D
      81       61.7237       4.68364       1.00000  0.000000E+00       1.00000  0.000000E+00      0.912718       59.7054       1.00000       9.74655  0.000000E+00     -0.444580  0.000000E+00     -0.444580  0.000000E+00  0.000000E+00      0.889159       57.3682      0.418910        1       Scatag_D
      81       1385.76       4939.37  0.000000E+00  0.000000E+00       2196.55  0.000000E+00       2390.40       6023.01  0.000000E+00       7836.15       42.4057       7089.84 -1.000000E+10 -1.000000E+10 -1.000000E+10 -1.000000E+10 -1.000000E+10  0.000000E+00  0.000000E+00  0.000000E+00  0.000000E+00  0.000000E+00  0.000000E+00  0.000000E+00  0.000000E+00  0.000000E+00  0.000000E+00  0.000000E+00  0.000000E+00       Funtag_D
      82  4.475382E+05  4.156283E+05       31909.9       7.12411      0.140243       1.31889      0.229356      0.233235     0.0568333     0.0264905    0.00704276      0.952872       Opttag_D
      82       61.7358       4.68371       1.00000  0.000000E+00       1.00000  0.000000E+00      0.912698       59.6990       1.00000       9.75433  0.000000E+00     -0.445503  0.000000E+00     -0.445503  0.000000E+00  0.000000E+00      0.891005       57.3682      0.418910        1       Scatag_D
      82       1386.64       4941.43  0.000000E+00  0.000000E+00       2197.38  0.000000E+00       2392.36       6024.06  0.000000E+00       7837.09       42.4262       7088.46 -1.000000E+10 -1.000000E+10 -1.000000E+10 -1.000000E+10 -1.000000E+10  0.000000E+00  0.000000E+00  0.000000E+00  0.000000E+00  0.000000E+00  0.000000E+00  0.000000E+00  0.000000E+00  0.000000E+00  0.000000E+00  0.000000E+00  0.000000E+00       Funtag_D
      83  4.475157E+05  4.155926E+05       31923.1       6.89363      0.140629       1.32713      0.229334      0.233218     0.0569266     0.0265004    0.00704386      0.953275       Opttag_D
      83       61.7493       4.68363       1.00000  0.000000E+00       1.00000  0.000000E+00      0.912676       59.6925       1.00000       9.76363  0.000000E+00     -0.446931  0.000000E+00     -0.446931  0.000000E+00  0.000000E+00      0.893861       57.3682      0.418910        1       Scatag_D
      83       1387.07       4945.61  0.000000E+00  0.000000E+00       2198.18  0.000000E+00       2394.74       6031.45  0.000000E+00       7837.89       42.5220       7085.63 -1.000000E+10 -1.000000E+10 -1.000000E+10 -1.000000E+10 -1.000000E+10  0.000000E+00  0.000000E+00  0.000000E+00  0.000000E+00  0.000000E+00  0.000000E+00  0.000000E+00  0.000000E+00  0.000000E+00  0.000000E+00  0.000000E+00  0.000000E+00       Funtag_D
      84  4.474956E+05  4.155435E+05       31952.1       10.8070      0.141187       1.33853      0.229302      0.233189     0.0570546     0.0265299    0.00704924      0.953868       Opttag_D
      84       61.7691       4.68377       1.00000  0.000000E+00       1.00000  0.000000E+00      0.912648       59.6834       1.00000       9.77716  0.000000E+00     -0.449062  0.000000E+00     -0.449062  0.000000E+00  0.000000E+00      0.898123       57.3682      0.418910        1       Scatag_D
      84       1389.19       4951.76  0.000000E+00  0.000000E+00       2199.24  0.000000E+00       2397.65       6050.04  0.000000E+00       7839.59       42.7207       7081.93 -1.000000E+10 -1.000000E+10 -1.000000E+10 -1.000000E+10 -1.000000E+10  0.000000E+00  0.000000E+00  0.000000E+00  0.000000E+00  0.000000E+00  0.000000E+00  0.000000E+00  0.000000E+00  0.000000E+00  0.000000E+00  0.000000E+00  0.000000E+00       Funtag_D
      85  4.474726E+05  4.155085E+05       31964.1       7.57258      0.141635       1.34726      0.229282      0.233167     0.0571458     0.0265533    0.00705371      0.954326       Opttag_D
      85       61.7852       4.68420       1.00000  0.000000E+00       1.00000  0.000000E+00      0.912629       59.6767       1.00000       9.78781  0.000000E+00     -0.450650  0.000000E+00     -0.450650  0.000000E+00  0.000000E+00      0.901299       57.3682      0.418910        1       Scatag_D
      85       1390.96       4956.52  0.000000E+00  0.000000E+00       2200.06  0.000000E+00       2399.29       6053.49  0.000000E+00       7841.56       42.8766       7079.33 -1.000000E+10 -1.000000E+10 -1.000000E+10 -1.000000E+10 -1.000000E+10  0.000000E+00  0.000000E+00  0.000000E+00  0.000000E+00  0.000000E+00  0.000000E+00  0.000000E+00  0.000000E+00  0.000000E+00  0.000000E+00  0.000000E+00  0.000000E+00       Funtag_D
      86  4.474490E+05  4.154724E+05       31976.6       7.22283      0.142131       1.35638      0.229265      0.233140     0.0572399     0.0265937    0.00705868      0.954775       Opttag_D
      86       61.8031       4.68484       1.00000  0.000000E+00       1.00000  0.000000E+00      0.912605       59.6684       1.00000       9.80034  0.000000E+00     -0.452365  0.000000E+00     -0.452365  0.000000E+00  0.000000E+00      0.904730       57.3682      0.418910        1       Scatag_D
      86       1392.92       4961.18  0.000000E+00  0.000000E+00       2200.88  0.000000E+00       2400.10       6058.09  0.000000E+00       7844.30       43.0580       7076.03 -1.000000E+10 -1.000000E+10 -1.000000E+10 -1.000000E+10 -1.000000E+10  0.000000E+00  0.000000E+00  0.000000E+00  0.000000E+00  0.000000E+00  0.000000E+00  0.000000E+00  0.000000E+00  0.000000E+00  0.000000E+00  0.000000E+00  0.000000E+00       Funtag_D
      87  4.474290E+05  4.154344E+05       31994.6       9.59761      0.142757       1.36737      0.229249      0.233117     0.0573391     0.0266016    0.00706567      0.955232       Opttag_D
      87       61.8253       4.68594       1.00000  0.000000E+00       1.00000  0.000000E+00      0.912571       59.6564       1.00000       9.81641  0.000000E+00     -0.454288  0.000000E+00     -0.454288  0.000000E+00  0.000000E+00      0.908576       57.3682      0.418910        1       Scatag_D
      87       1395.68       4965.94  0.000000E+00  0.000000E+00       2201.87  0.000000E+00       2400.71       6066.75  0.000000E+00       7848.37       43.2549       7072.06 -1.000000E+10 -1.000000E+10 -1.000000E+10 -1.000000E+10 -1.000000E+10  0.000000E+00  0.000000E+00  0.000000E+00  0.000000E+00  0.000000E+00  0.000000E+00  0.000000E+00  0.000000E+00  0.000000E+00  0.000000E+00  0.000000E+00  0.000000E+00       Funtag_D
      88  4.474103E+05  4.154090E+05       32001.3       6.79108      0.143227       1.37508      0.229239      0.233101     0.0574052     0.0266086    0.00706932      0.955507       Opttag_D
      88       61.8415       4.68678       1.00000  0.000000E+00       1.00000  0.000000E+00      0.912539       59.6464       1.00000       9.82926  0.000000E+00     -0.455616  0.000000E+00     -0.455616  0.000000E+00  0.000000E+00      0.911232       57.3682      0.418910        1       Scatag_D
      88       1397.12       4968.79  0.000000E+00  0.000000E+00       2202.59  0.000000E+00       2400.79       6068.07  0.000000E+00       7851.74       43.3773       7068.82 -1.000000E+10 -1.000000E+10 -1.000000E+10 -1.000000E+10 -1.000000E+10  0.000000E+00  0.000000E+00  0.000000E+00  0.000000E+00  0.000000E+00  0.000000E+00  0.000000E+00  0.000000E+00  0.000000E+00  0.000000E+00  0.000000E+00  0.000000E+00       Funtag_D
      89  4.473932E+05  4.153910E+05       32002.2       6.16289      0.143599       1.38061      0.229237      0.233094     0.0574521     0.0265971    0.00706960      0.955635       Opttag_D
      89       61.8541       4.68745       1.00000  0.000000E+00       1.00000  0.000000E+00      0.912506       59.6367       1.00000       9.84037  0.000000E+00     -0.456704  0.000000E+00     -0.456704  0.000000E+00  0.000000E+00      0.913407       57.3682      0.418910        1       Scatag_D
      89       1397.23       4970.13  0.000000E+00  0.000000E+00       2203.10  0.000000E+00       2400.24       6068.69  0.000000E+00       7854.38       43.4455       7064.98 -1.000000E+10 -1.000000E+10 -1.000000E+10 -1.000000E+10 -1.000000E+10  0.000000E+00  0.000000E+00  0.000000E+00  0.000000E+00  0.000000E+00  0.000000E+00  0.000000E+00  0.000000E+00  0.000000E+00  0.000000E+00  0.000000E+00  0.000000E+00       Funtag_D
      90  4.473757E+05  4.153738E+05       32002.0       7.72472      0.144049       1.38662      0.229233      0.233088     0.0574971     0.0265715    0.00706944      0.955740       Opttag_D
      90       61.8691       4.68845       1.00000  0.000000E+00       1.00000  0.000000E+00      0.912462       59.6259       1.00000       9.85357  0.000000E+00     -0.457940  0.000000E+00     -0.457940  0.000000E+00  0.000000E+00      0.915881       57.3682      0.418910        1       Scatag_D
      90       1397.17       4971.22  0.000000E+00  0.000000E+00       2203.71  0.000000E+00       2399.44       6069.88  0.000000E+00       7857.43       43.4991       7059.60 -1.000000E+10 -1.000000E+10 -1.000000E+10 -1.000000E+10 -1.000000E+10  0.000000E+00  0.000000E+00  0.000000E+00  0.000000E+00  0.000000E+00  0.000000E+00  0.000000E+00  0.000000E+00  0.000000E+00  0.000000E+00  0.000000E+00  0.000000E+00       Funtag_D
      91  4.473572E+05  4.153551E+05       32002.1       9.32882      0.144695       1.39465      0.229229      0.233081     0.0575459     0.0265408    0.00707025      0.955965       Opttag_D
      91       61.8905       4.69032       1.00000  0.000000E+00       1.00000  0.000000E+00      0.912413       59.6134       1.00000       9.87042  0.000000E+00     -0.459588  0.000000E+00     -0.459588  0.000000E+00  0.000000E+00      0.919176       57.3682      0.418910        1       Scatag_D
      91       1397.49       4973.56  0.000000E+00  0.000000E+00       2204.57  0.000000E+00       2398.56       6069.68  0.000000E+00       7861.75       43.5580       7052.99 -1.000000E+10 -1.000000E+10 -1.000000E+10 -1.000000E+10 -1.000000E+10  0.000000E+00  0.000000E+00  0.000000E+00  0.000000E+00  0.000000E+00  0.000000E+00  0.000000E+00  0.000000E+00  0.000000E+00  0.000000E+00  0.000000E+00  0.000000E+00       Funtag_D
      92  4.473369E+05  4.153443E+05       31992.7       7.20055      0.145204       1.40014      0.229224      0.233079     0.0575740     0.0265223    0.00707051      0.956040       Opttag_D
      92       61.9071       4.69202       1.00000  0.000000E+00       1.00000  0.000000E+00      0.912377       59.6052       1.00000       9.88289  0.000000E+00     -0.460797  0.000000E+00     -0.460797  0.000000E+00  0.000000E+00      0.921593       57.3682      0.418910        1       Scatag_D
      92       1397.59       4974.34  0.000000E+00  0.000000E+00       2205.19  0.000000E+00       2397.64       6062.24  0.000000E+00       7864.74       43.6038       7047.31 -1.000000E+10 -1.000000E+10 -1.000000E+10 -1.000000E+10 -1.000000E+10  0.000000E+00  0.000000E+00  0.000000E+00  0.000000E+00  0.000000E+00  0.000000E+00  0.000000E+00  0.000000E+00  0.000000E+00  0.000000E+00  0.000000E+00  0.000000E+00       Funtag_D
      93  4.473156E+05  4.153387E+05       31976.9       6.86649      0.145853       1.40650      0.229224      0.233084     0.0575885     0.0264950    0.00707245      0.956111       Opttag_D
      93       61.9281       4.69474       1.00000  0.000000E+00       1.00000  0.000000E+00      0.912343       59.5978       1.00000       9.89640  0.000000E+00     -0.461934  0.000000E+00     -0.461934  0.000000E+00  0.000000E+00      0.923868       57.3682      0.418910        1       Scatag_D
      93       1398.36       4975.08  0.000000E+00  0.000000E+00       2205.92  0.000000E+00       2396.46       6048.39  0.000000E+00       7868.45       43.6906       7040.53 -1.000000E+10 -1.000000E+10 -1.000000E+10 -1.000000E+10 -1.000000E+10  0.000000E+00  0.000000E+00  0.000000E+00  0.000000E+00  0.000000E+00  0.000000E+00  0.000000E+00  0.000000E+00  0.000000E+00  0.000000E+00  0.000000E+00  0.000000E+00       Funtag_D
      94  4.473029E+05  4.153344E+05       31968.4       11.6545      0.146720       1.41430      0.229231      0.233091     0.0575996     0.0264682    0.00707623      0.956349       Opttag_D
      94       61.9555       4.69846       1.00000  0.000000E+00       1.00000  0.000000E+00      0.912300       59.5874       1.00000       9.91393  0.000000E+00     -0.463065  0.000000E+00     -0.463065  0.000000E+00  0.000000E+00      0.926129       57.3682      0.418910        1       Scatag_D
      94       1399.85       4977.56  0.000000E+00  0.000000E+00       2206.84  0.000000E+00       2394.99       6038.79  0.000000E+00       7873.42       43.8553       7033.14 -1.000000E+10 -1.000000E+10 -1.000000E+10 -1.000000E+10 -1.000000E+10  0.000000E+00  0.000000E+00  0.000000E+00  0.000000E+00  0.000000E+00  0.000000E+00  0.000000E+00  0.000000E+00  0.000000E+00  0.000000E+00  0.000000E+00  0.000000E+00       Funtag_D
      95  4.472866E+05  4.153359E+05       31950.7       5.78789      0.146852       1.41490      0.229234      0.233099     0.0575957     0.0264469    0.00707602      0.956201       Opttag_D
      95       61.9597       4.69918       1.00000  0.000000E+00       1.00000  0.000000E+00      0.912294       59.5858       1.00000       9.91589  0.000000E+00     -0.463046  0.000000E+00     -0.463046  0.000000E+00  0.000000E+00      0.926091       57.3682      0.418910        1       Scatag_D
      95       1399.77       4976.02  0.000000E+00  0.000000E+00       2206.89  0.000000E+00       2394.46       6024.91  0.000000E+00       7873.72       43.9169       7031.00 -1.000000E+10 -1.000000E+10 -1.000000E+10 -1.000000E+10 -1.000000E+10  0.000000E+00  0.000000E+00  0.000000E+00  0.000000E+00  0.000000E+00  0.000000E+00  0.000000E+00  0.000000E+00  0.000000E+00  0.000000E+00  0.000000E+00  0.000000E+00       Funtag_D
      96  4.472744E+05  4.153366E+05       31937.7       5.31307      0.147060       1.41584      0.229238      0.233108     0.0575938     0.0264137    0.00707584      0.956138       Opttag_D
      96       61.9663       4.70018       1.00000  0.000000E+00       1.00000  0.000000E+00      0.912282       59.5825       1.00000       9.91951  0.000000E+00     -0.463024  0.000000E+00     -0.463024  0.000000E+00  0.000000E+00      0.926048       57.3682      0.418910        1       Scatag_D
      96       1399.70       4975.37  0.000000E+00  0.000000E+00       2206.97  0.000000E+00       2393.75       6015.59  0.000000E+00       7874.27       44.0157       7028.06 -1.000000E+10 -1.000000E+10 -1.000000E+10 -1.000000E+10 -1.000000E+10  0.000000E+00  0.000000E+00  0.000000E+00  0.000000E+00  0.000000E+00  0.000000E+00  0.000000E+00  0.000000E+00  0.000000E+00  0.000000E+00  0.000000E+00  0.000000E+00       Funtag_D
      97  4.472615E+05  4.153355E+05       31926.0       6.27290      0.147509       1.41835      0.229243      0.233125     0.0575968     0.0263514    0.00707602      0.956161       Opttag_D
      97       61.9803       4.70212       1.00000  0.000000E+00       1.00000  0.000000E+00      0.912253       59.5754       1.00000       9.92829  0.000000E+00     -0.463331  0.000000E+00     -0.463331  0.000000E+00  0.000000E+00      0.926662       57.3682      0.418910        1       Scatag_D
      97       1399.77       4975.60  0.000000E+00  0.000000E+00       2207.28  0.000000E+00       2392.79       6006.89  0.000000E+00       7876.02       44.1434       7023.51 -1.000000E+10 -1.000000E+10 -1.000000E+10 -1.000000E+10 -1.000000E+10  0.000000E+00  0.000000E+00  0.000000E+00  0.000000E+00  0.000000E+00  0.000000E+00  0.000000E+00  0.000000E+00  0.000000E+00  0.000000E+00  0.000000E+00  0.000000E+00       Funtag_D
      98  4.472509E+05  4.153366E+05       31914.3       14.9650      0.148609       1.42324      0.229260      0.233169     0.0575940     0.0261775    0.00707656      0.956402       Opttag_D
      98       62.0137       4.70705       1.00000  0.000000E+00       1.00000  0.000000E+00      0.912183       59.5591       1.00000       9.94888  0.000000E+00     -0.464025  0.000000E+00     -0.464025  0.000000E+00  0.000000E+00      0.928051       57.3682      0.418910        1       Scatag_D
      98       1399.98       4978.11  0.000000E+00  0.000000E+00       2208.04  0.000000E+00       2390.20       6000.28  0.000000E+00       7880.20       44.3601       7013.18 -1.000000E+10 -1.000000E+10 -1.000000E+10 -1.000000E+10 -1.000000E+10  0.000000E+00  0.000000E+00  0.000000E+00  0.000000E+00  0.000000E+00  0.000000E+00  0.000000E+00  0.000000E+00  0.000000E+00  0.000000E+00  0.000000E+00  0.000000E+00       Funtag_D
      99  4.472301E+05  4.153387E+05       31891.4       5.93868      0.149007       1.42494      0.229264      0.233182     0.0575884     0.0261191    0.00707503      0.956323       Opttag_D
      99       62.0256       4.70894       1.00000  0.000000E+00       1.00000  0.000000E+00      0.912159       59.5541       1.00000       9.95597  0.000000E+00     -0.464337  0.000000E+00     -0.464337  0.000000E+00  0.000000E+00      0.928675       57.3682      0.418910        1       Scatag_D
      99       1399.38       4977.28  0.000000E+00  0.000000E+00       2208.35  0.000000E+00       2389.34       5980.95  0.000000E+00       7881.80       44.3804       7009.94 -1.000000E+10 -1.000000E+10 -1.000000E+10 -1.000000E+10 -1.000000E+10  0.000000E+00  0.000000E+00  0.000000E+00  0.000000E+00  0.000000E+00  0.000000E+00  0.000000E+00  0.000000E+00  0.000000E+00  0.000000E+00  0.000000E+00  0.000000E+00       Funtag_D

   MaxLik Scaling Cycle 0106

   Wilson plot

   Statistics in resolution bins after refinement cycle 0003
```


|  |  |
| --- | --- |
|  | - Rxpct factors plot- Rxpct factors table- <Log-Lik Gain>- Ln Sigma A- Correlation Coefficients |


```
   Iters     Tot_Funct    Xray_Funct    Geom_Funct          Grms     rmsD2init     maxD2init             R         Rfree           LLG       LLGfree     rms(bond)    rms(angle)
   Iters             K   B_IMPF_FRAG        K_MISS   B_TEMP_MISS   K_IMPF_MISS   B_IMPF_MISS        K_SOLV   B_TEMP_SOLV   K_IMPF_SOLV   B_IMPF_SOLV        B_TEMP     B_TEMP_11     B_TEMP_12     B_TEMP_22     B_TEMP_13     B_TEMP_23     B_TEMP_33      K_WILSON      B_WILSON      FGCYCLES
   Iters          BOND         ANGLE      IMPROPER       TORSION    SINTORSION      TRIGONAL        PLANAR  BCORRELATION       CONTACT     IDEALDIST        CHIRAL    SIMILARITY       OCCSSUM  UTILDISTANCE     UTILANGLE   UTILTORSION        RHOFIT        ADP-01        ADP-02        ADP-03        ADP-04        ADP-05        ADP-06        ADP-07        ADP-08        ADP-09        ADP-10      RHOFITPC            QM
     100  4.472200E+05  4.153411E+05       31878.9       4.60659      0.149239       1.42531      0.229266      0.233190     0.0575823     0.0260838    0.00707237      0.956331       Opttag_D
     100       62.0326       4.71016       1.00000  0.000000E+00       1.00000  0.000000E+00      0.912146       59.5518       1.00000       9.95954  0.000000E+00     -0.464526  0.000000E+00     -0.464526  0.000000E+00  0.000000E+00      0.929053       57.3682      0.418910        1       Scatag_D
     100       1398.32       4977.37  0.000000E+00  0.000000E+00       2208.51  0.000000E+00       2388.34       5972.63  0.000000E+00       7882.35       44.3184       7007.08 -1.000000E+10 -1.000000E+10 -1.000000E+10 -1.000000E+10 -1.000000E+10  0.000000E+00  0.000000E+00  0.000000E+00  0.000000E+00  0.000000E+00  0.000000E+00  0.000000E+00  0.000000E+00  0.000000E+00  0.000000E+00  0.000000E+00  0.000000E+00       Funtag_D
   Geometry Restraint Deviations SCREEN Information

 

 The refinement has NOT converged.

 No -bcorrel_zeroocchydrogens_factor flag found - keeping default: will set B for OCC=0 hydrogens to    1.200 times heavy-atom B


 TLS information. Total number of groups:     2


 Group   Natoms    FixRB   FixTLS   Group Tag    Group Spec
     1     2768      Y        Y     tls1    { A|* }
     2     2758      Y        Y     tls2    { B|* }
 
NOTE BUSTER_TLS_SET tls1 { A|* }
NOTE BUSTER_TLS_T   tls1     -1.64775E-01 -3.36840E-02  2.15737E-01 -3.93502E-02 -3.35143E-02 -2.21740E-02
NOTE BUSTER_TLS_L   tls1      1.33783E+00  1.61817E+00  1.44183E+00 -3.13773E-01  8.90801E-01 -8.51731E-01
NOTE BUSTER_TLS_S   tls1      1.51047E-01 -2.84462E-01  3.13261E-01  6.85951E-02 -1.30648E-01 -2.56570E-01  7.59762E-02  1.23108E-01
NOTE BUSTER_TLS_O   tls1      2.31588E+01 -6.71772E+01  1.06797E+01
NOTE BUSTER_TLS_FIX tls1 ALL
 
NOTE BUSTER_TLS_SET tls2 { B|* }
NOTE BUSTER_TLS_T   tls2     -2.34737E-01 -9.54492E-02  3.04843E-01  1.30665E-02 -3.15132E-02  3.78123E-02
NOTE BUSTER_TLS_L   tls2      1.51690E+00  1.05873E+00  1.03399E+00  6.62845E-01  9.72762E-01  7.91924E-01
NOTE BUSTER_TLS_S   tls2      8.67167E-04  4.13351E-02 -2.05843E-01 -1.01273E-01 -1.11597E-01  4.88749E-02  1.27152E-01 -6.61129E-02
NOTE BUSTER_TLS_O   tls2     -2.22253E+01 -7.89989E+01  3.21640E+00
NOTE BUSTER_TLS_FIX tls2 ALL

 For final position, Geometry function summary
     term                           number     weight       rms        GooF      function     fn/numb
     ================================================================================================
     BOND bond lengths (angs)       5642       2.000      0.0071       0.352      1398.32       0.248
     ANGL bond angles (degs)        7656       2.000        0.96       0.570      4977.37       0.650
     TORS split-harmonic tors       1266       0.000       16.97       1.068         0.00       0.000
     SINTOR sinusoidal tors         1266       2.000       16.97                  2208.51       1.744
     PLAN general plane (angs)       958       5.000      0.0141       0.706      2388.34       2.493
     BCORrelations (angs^2)         5642      10.000       0.912       0.325      5972.63       1.059
     CONT (bad contacts)               0       5.000                                 0.00
     IDEAL(ideal-dist contact)      3897       4.000                              7882.35
     CHIRAL (gelly semiharmon)       742       5.000                                44.32
     SIM similarity restraint for NCS and/or TARGET                               7007.08
     OCCSUM restraint on sum of occupanies                                           0.00       as none defined
     DISTAN utility restraints interatomic distance                                  0.00       as none defined
     UTILANGLE utility angle restraints                                              0.00       as none defined
     UTILTOR utility restraints on torsion angles                                    0.00       as none defined
     Peptide omega torsion angles                           2.86 degs - monitor only
 
 WARNING: Have |delta/sigma| deviations > 5.0 sigma. Number of outliers for each term: 
 WARNING:         2 bond angles.  Worst is     5.6 sigs   104.81 degs A|27:CA(ILE)=A|27:C(ILE)=A|28:N.B(ASN)

 Weighted rms      0.0071         0.956  (temporary rms bond and angle for BUSTER to pick up!)
 Weighted intra-residual rms(bond) for StdProtein (excluding H)      0.0068
 Outputing geometry progress graph in plotmtv format to file:Geom_Res.mtv

 ############### Running final geometry/screen analysis (screen_final.txt)

 s/r gelly normal termination
```

Statistics and scale factors vs. cycle number
```

|  |  |  |
| --- | --- | --- |
|  | - Rxpct factors- <Log-Lik Gain>- GEOM Residuals- Ln Sigma_A_ | - Scale Factors- B Factors- Imperfection Factors |

```
   Final Geometry Information


---


### 2. Final results

Statistics and scale factors vs. cycle number
```

|  |  |  |
| --- | --- | --- |
|  | - Rxpct factors- <Log-Lik Gain>- GEOM Residuals- Ln Sigma_A_ | - Scale Factors- B Factors- Imperfection Factors |

```
   Average Figure of Merit
   Structure Factor Amplitudes


 # normal termination

  Program stopped.
```

closesok message: time spent in tree = 1297.84 (user) 39.13 (sys)

---

# Normal termination

```
 Resources usage summary:
     Time spent in client ...................  1297.84 (user)       39.16 (system)
     Time spent in scripts (    27 calls) ...     2.26 (user)        0.14 (system)
     Time spent in server ...................     0.00 (user)        0.00 (system)

     Max resident memory used (kb) .......... 431028
     Number of page faults .................. 0 (major)    616194 (minor)
     Number of context switches ............. 1976 (niced)    5290 (other)
```
